# Precision fabrication of polymer nanostructures on recyclable DNA template

**DOI:** 10.1002/smo.20240006

**Published:** 2024-06-11

**Authors:** Zi'an Lin, Xuemei Xu, Yiwei Shi, Yuzhou Wu

**Affiliations:** ^1^ Hubei Key Laboratory of Bioinorganic Chemistry & Materia Medica School of Chemistry and Chemical Engineering Hubei Engineering Research Center for Biomaterials and Medical Protective Materials Huazhong University of Science and Technology (HUST) Wuhan China; ^2^ Key Laboratory for Green Chemical Process of Ministry of Education Hubei Key Lab of Novel Reaction & Green Chemical Technology School of Chemical Engineering and Pharmacy Wuhan Institute of Technology Wuhan China

**Keywords:** DNA origami, nano‐patterned polymers, photo‐regulation

## Abstract

The fabrication of precisely patterned polymers at the nanoscale is of critical importance. We have previously succeeded in creating various nanopatterned polymers with nanoscale resolution through the use of in situ atom transfer radical polymerization (ATRP) techniques on deoxyribonucleic acid (DNA) origami. However, separating nanopatterned polymers from the origami template without damaging the origami presents a significant challenge, thereby increasing costs and limiting the development of applications involving nanopatterned polymers. Here, we achieved spatially and temporally controlled release of DNA origami templates through photo‐regulation by incorporating azobenzene‐modified DNA into the initiator. Under UV exposure, azobenzene isomerization rapidly induces the disassociation of patterned polymers from the origami template at ambient temperatures, without damaging the DNA origami. Additionally, the released origami template can be reused as a template for the cyclic production of nanopatterned polymers. This method provides a pathway for the large‐scale production of patterned polymers at reduced costs and facilitates dynamic control over the polymer‐DNA complex, with potential applications in both the biomedical and chemical fields.

## Graphical abstract

The fabrication of precisely patterned polymers at nanoscale is of critical importance. We have previously succeeded in creating various nanopatterned polymers with nanoscale resolution through the use of in situ atom transfer radical polymerization techniques on deoxyribonucleic acid (DNA) origami. However, separating nanopatterned polymers from the origami template without damaging the origami presents a significant challenge, thereby increasing costs and limiting the development of application involving nanopatterned polymers. Here, we achieved spatially and temporally controlled release of DNA origami templates through photo‐regulation by incorporating azobenzene‐modified DNA into the initiator. Under UV exposure, azobenzene isomerization rapidly induces the disassociation of patterned polymers from the origami template at ambient temperature, without damaging the DNA origami. Additionally, the released origami template can be reused as a template for the cyclic production of nanopatterned polymers. This method provides a pathway for the large‐scale production of patterned polymers at reduced costs and facilitates dynamic control over the polymer‐DNA complex, with potential application in both the biomedical and chemical fields.

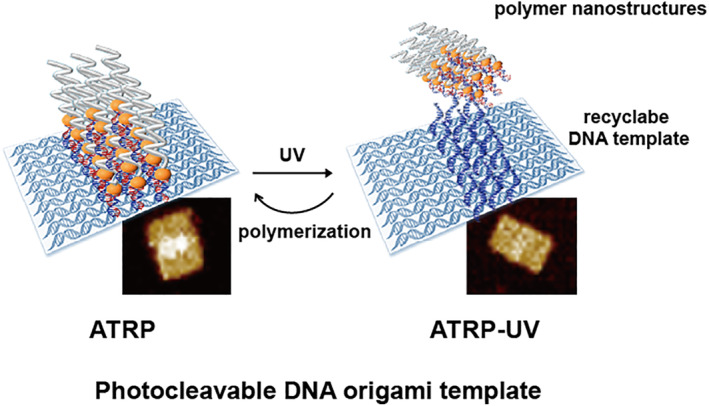

## 1 INTRODUCTION

Nanofabrication of functional polymers with high precision and resolution is in high demand for a variety of potential applications, including biochips for cell adhesion control, micro/nanofluidic systems, and photonic materials.[^1^, ^2^, ^3^] However, the currently available techniques are primarily based on top‐down strategies, such as lithography, which are costly and time‐consuming. Recently developed bottom‐up strategies offer a new opportunity for the fabrication of nano‐patterned polymers, assisted by deoxyribonucleic acid (DNA) origami nanotechnology, which enables controlled shaping at nanometer resolution.[^4^, ^5^, ^6^, ^7^]

DNA origami represents a powerful tool for the systematic design of DNA nanostructures through the self‐assembly of scaffold DNA and staple DNA sequences. Each DNA sequence within the defined nanostructure encodes a specific position, allowing for precise and addressable modifications.^[8]^ This tool has been widely utilized to organize nanoparticles, proteins, and small functional molecules with nanometer resolution.[^9^, ^10^, ^11^, ^12^, ^13^, ^14^, ^15^] Previously, we reported on the “grafting from” method for spatially controlling polymer growth on the surface of DNA origami utilizing atom transfer radical polymerization (ATRP).^[4]^ Briefly, an initiator was incorporated into DNA strands and introduced to hybridize with DNA origami at predefined positions, subsequently inducing the ATRP reaction to grow nano‐patterned polymers on the DNA origami. We have successfully established a DNA‐templated strategy for the growth of various polymers with distinct shapes and found that the anisotropic distribution of these polymers at nanometer resolution can significantly affect cell behavior, including cell uptake efficiency and penetration depth.^[16]^ However, the resulting patterned polymers are tightly bound to the DNA origami template through Watson‐Crick base pairing. Separating the origami template from the nano‐patterned polymers is challenging, especially without causing harm to the DNA origami template.[^4^, ^5^, ^6^, ^16^] This increases the cost and hinders the development of such nano‐patterned polymers.

**Scheme 1:**
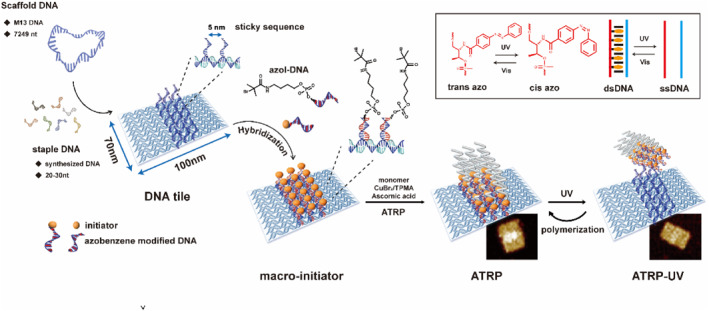
Schematic of the azobenzene modified DNA origami hybrids with polymers, initiator and azobenzene were modified in one DNA strand and were incorporated in DNA origami initiating ATRP reaction for growth of nano‐patterned polymers in predesigned nano‐scale site, then under the UV exposure, azobenzene isomerizes from trans to cis form causing the disassociation of double strand DNA leading to the separation of DNA origami template and polymer layers. ATRP, atom transfer radical polymerization; DNA, deoxyribonucleic acid

Herein, we have developed a photoregulation strategy that achieved complete separation of the DNA origami template from polymers with spatial and temporal control, without harm to the origami template. Furthermore, we demonstrate the universality of this strategy by reusing the separated DNA origami template through the incorporation of photoresponsive azobenzene molecules into the initiator DNA (Scheme 1). Azobenzene's photodynamic configuration has been extensively studied, and the molecule serves as a prototypical photo‐regulator.[^17^, ^18^] Upon exposure to UV light, azobenzene isomerizes from its trans to cis form through the rotation of its *N* = *N* bonds, with the trans form adopting a planar and nonpolar configuration conducive to intercalation with other planar components, such as nucleotide bases. The cis form, however, becomes nonplanar and polar, resulting in steric hindrance with those components.^[19]^ This dynamic configuration change of azobenzene has applications in various fields, including as a reaction modulator, nanotweezers, and dynamically regulating binding properties.[^20^, ^21^, ^22^] Incorporating azobenzene into the DNA endows the molecule with photoresponsiveness. Upon UV exposure, the configurational change of azobenzene interferes with the stability of double‐stranded DNA, ultimately leading to disassociation.[^20^, ^23^, ^24^, ^25^] Choosing azobenzene as the photo‐regulator, we have systematically investigated the properties of azobenzene‐tethered DNA under various conditions. This enabled the efficient and rapid separation of the origami template from nano‐patterned polymers. Most importantly, the separated DNA origami template could be directly reused in subsequent polymerizations without any interference. This study provides dynamic spatial and temporal control over the release of nano‐patterned polymers from the DNA origami template, facilitated by light regulation.

## 2 RESULTS AND DISCUSSION

**FIGURE 1:**
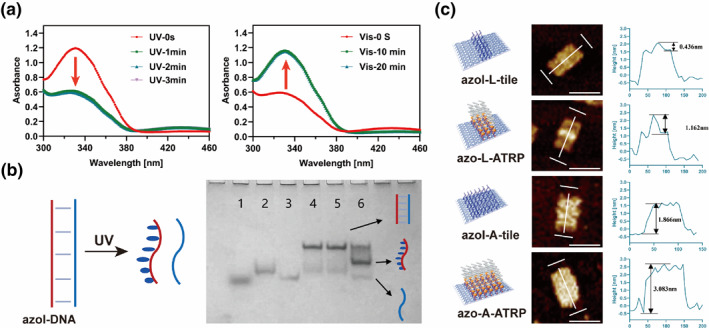
(a) Kinetic study of photoisomerization of azobenzene‐modified DNA under UV (left) or Vis (right). (b) 12% native PAGE characterization of the photo‐regulated association/disassociation of azoI‐DNA/sticky DNA by UV light. Lane 1: loading buffer; lane 2: azoI‐DNA; lanes 3: sticky DNA; lanes 4: azoI‐DNA/sticky DNA; lanes 5: azoI‐DNA/sticky at 37°C without UV; lane 6: azoI‐DNA/sticky at 37°C under UV exposure. (c) AFM images and height profiles of azoI‐L‐tile, azoI‐L‐ATRP, azoI‐A‐tile, azoI‐A‐ATRP. Scale bars: 100 nm. AFM, atomic force microscopy; ATRP, atom transfer radical polymerization; DNA, deoxyribonucleic acid, Vsi, visible.

In a proof‐of‐concept experiment demonstrating the photoregulation of azobenzene‐modified DNA behavior within DNA origami, we first explored the photoswitching properties of azobenzene‐modified single‐stranded oligonucleotides. Here, we utilized a sequence‐complementary azoI‐DNA/sticky DNA (azoI‐DNA: ICTCXTAXCCXACXCTXACXTA; Sticky DNA: TAGTAGGTGGTAGAG; where X stands for azobenzene and I for the initiator) as the fundamental photoresponsive module. To experimentally investigate the isomerization rate and efficiency of azobenzene‐modified DNA, the UV‐Vis spectrum of azoI‐DNA was measured at various times, under differing light intensities, and temperatures (Figure 1a, Figure S1). The absorbance of azobenzene at 340 nm significantly decreased within 2 min under UV exposure (365 nm) and recovered within 10 min under visible (Vis) light treatment (450 nm). Light intensity of UV irradiation and temperature can influence the isomerization efficiency of azobenzene; for instance, 10 mW/cm^2^ UV light intensity led to a 26.5% reduction in absorbance, while 100 mW/cm^2^ increased it to 38.4%. On the other hand, higher temperature seems to be preferred, but high temperature could also interrupt the DNA origami structure. Considering all of these factors, we selected 10 min at 60–70 mW/cm^2^ and 37°C as the experimental conditions. These results collectively confirmed the rapid photoresponsive property of azobenzene‐modified DNA. We then investigated the photoresponsive DNA hybridization properties of azobenzene‐modified dsDNA in bulk solution using native polyacrylamide gel electrophoresis. As shown in Figure 1b, azoI‐DNA could hybrid with sticky DNA forming the DNA pair, under theVis light, no changes happened while the intensity of the double‐stranded DNA band decreased as the unbound single‐stranded DNA band increased after irradiation under the UV light at 365 nm. Here, we found that not all double‐stranded DNA bands disappeared; a number of them remained. We hypothesized that this occurs because we incorporated the azobenzene molecule into only one strand, not both. Upon dissociation by UV irradiation, a high binding affinity remains between the two strands, allowing some single‐stranded DNA to rehybridize during the electrophoresis process.[^17^, ^18^]

After confirming the photoresponsive property of azoI‐DNA, we assembled it into DNA origami and initiated in situ atom‐transfer radical polymerization. A 2D rectangular DNA origami sheet, measuring 100 × 70 nm^2^ with a thickness of 2 nm, was employed as the template to facilitate the growth of nano‐patterned polymers, as we previously reported,[^17^, ^18^] to achieve precise and shape‐controlled polymers. Briefly, an additional sequence of 15 nt (sticky DNA) was elongated from the 3′ end of the surface of DNA origami (Scheme 1), complementary to azoI‐DNA modified with an initiator, to form the DNA origami macroinitiator, enabling atom‐transfer radical polymerization under specific conditions. Monomer of poly (ethylene glycol) methyl ether methacrylate (PEGMEMA) was selected due to its biocompatibility and wide applications in bionanotechnology,^[27]^ polymerizing at predefined positions as designed, with PEG dimethacrylate added as the cross‐linker. The in situ ATRP reaction was conducted in 1×Tris‐acetate‐EDTA buffer at pH 8.

Two different patterns were selected for concern: L‐tile (with four lines arranged in the center) and A‐tile (with 11 lines arranged to form a rectangular pattern) (Figure 1c) to characterize the synthesis of bottom‐up fabricated polymers. Atomic force microscopy (AFM) characterization provided visualization of the surface pattern morphology of these DNA origami, including details of height data before and after the polymer reaction, with monomer/initiator ratios of 8000:1. It was observed that both L‐tile macroinitiator and A‐tile macroinitiator exhibited a thinner layer coverage before the reaction, and the A‐tile ATRP origami extended beyond the original structure; additionally, the margins became blurred and rough (Figure 1c), suggesting successful synthesis of patterned polymers. According to the height profile analysis, the average height of azoI‐L‐tile increased by 0.73 nm compared to the L‐tile macroinitiator in the center area; a 1.22 nm height increase was observed between azoI‐A‐tile and A‐tile macroinitiator. Correlating with previously published data. However, the increased height remained significantly smaller than the distances between adjacent initiator positions (5.8 nm), indicating that the polymers were likely to adopt a mushroom‐like collapsed structure.[^17^, ^18^] Consequently, denser patterned polymers were successfully constructed using this bottom‐up ATRP strategy at the predefined positions on DNA origami, the modification of azobenzene molecules into the initiator strands did not interfere with the overall reaction process.

**FIGURE 2:**
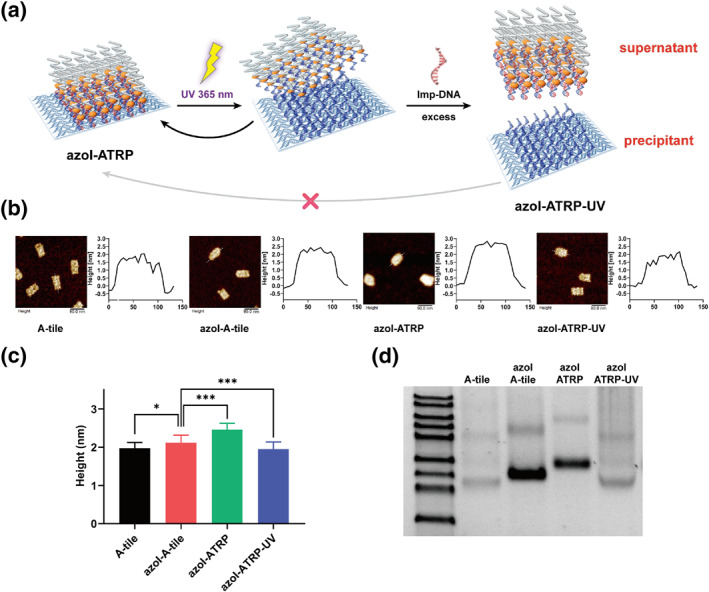
(a) photoresponsiveness of azobenzene tethered DNA origami for separation of the origami template. (b) AFM images and height profiles of A‐tile, azoI‐tile, azoI‐ATRP, azoI‐ATRP‐UV. Scale bars: 90 nm. (c) average height analysis of A tile, azoI‐tile, azoI‐ATRP and azoI‐ATRP‐UV under the UV irradiation. Data represent means ± SE, *n* = 30. *: *p* < 0.01. ***: *p* < 0.005. (d) 2% agarose gel characterization of separation of polymer layers. Lane 1: A‐tile; lane 2: azoI‐tile; lane 3: azoI‐ATRP; lane 4: azoI‐ATRP‐UV. AFM, atomic force microscopy; ATRP, atom transfer radical polymerization; DNA, deoxyribonucleic acid.

Subsequent to the polymerization reaction, photoirradiation under UV exposure at 365 nm is expected to trigger the transformation of azobenzene from the trans‐form to the cis‐form, immediately disrupting the interaction between complementary strands and inducing dissociation of double strands into single strands. The UV‐sensitive azobenzene molecules, which were introduced alongside initiators to attach to polymers after the ATRP reaction, could detach from the DNA origami, facilitating the separation of patterned polymers from the DNA origami template. We first tested the stability of DNA origami under UV exposure, at a light intensity of 60–70 mW/cm^2^ and 37°C for 10 min (Figure S2). AFM images showed that after the UV irradiation, DNA origami could still keep its intact structure without significant harm.[^17^, ^18^] In order to better characterize whether the patterned polymers could be separated with DNA origami, a higher polymer coverage pattern (A‐tile) was chosen. Afterwards, the PEGMEMA polymerized azoI‐tile was synthesized as describe above. The same condition was applied to the azoI‐tile, remaining solutions were immediately collected in an attempt to separate the patterned polymers and DNA origami templates by PEG purification (see methods in details).^[30]^ However, no difference was observed between the origami collected before and after UV irradiation (Figure S3). Running a 2% agarose gel revealed that the azoI ‐ATRP‐UV sample was at the same level as the azoI‐ATRP sample, indicating no change in molecular weight; similarly, height analysis showed no statistical difference (Figure S3). We assumed that UV irradiation disrupted the interaction of double strands and caused their dissociation, but this was a temporary separation, the departed single strand DNA will rapidly convert to pairing state, particularly once UV exposure was halted, even in the absence of Vis light.[^20^, ^21^, ^22^] To overcome this, a set of impeding strands (imp‐DNA), fully complementary to azoI‐DNA strands and identical to sticky‐DNA (Figure 2a), was introduced into the system upon UV light exposure. At the initial time of UV irradiation, azobenzene molecules changed from trans to cis and interrupt the dsDNA linked between polymer layers and origami templates, the imp‐DNA competed with the DNA origami template for rebinding to azoI‐DNA, with a 10 folds excess of imp‐DNA added to the solution, azoI‐DNA is more likely to interact with imp‐DNA than with the DNA origami template, regardless of preference. We first tested if the imp‐DNA would directly compete azoI‐DNA attaching to polymers with DNA origami template in the absence of UV light, it showed no difference after adding imp‐DNA into azoI‐ATRP sample although the imp‐DNA's concentration is much higher than origami template (Figure S4), due to the inner binding site for imp‐DNA was hidden by densely patterned polymers. Through this approach, a discernible difference was successfully observed after UV exposure. Running a 2% agarose gel showed that the azoI‐A‐ATRP band migrates more slowly and settled higher in the gel compared to the UV one (Figure 2d), representing the change in molecular weight of azoI‐ATRP‐UV. The AFM assay also confirmed our findings. In 2D views, A‐tile provided a plain image, with only those areas containing sticky strands standing out slightly, offering a subtle contrast to neighboring sites (Figure 2b). Upon addition of the initiator, the hydrogen bonds between two single‐stranded DNAs provided a more robust structure, enabling the sticky strands to be more pronounced. And after the ATRP reaction, the edge of azoI‐ATRP was much tougher, and the surface of the origami also became unclear, it was most likely due to the growth of the polymer layer, but when it was exposed under the UV light, the appearance of azoI‐ATRP‐UV return back to the original A‐tile indicate the polymer layer was left. The height of the bare A‐tile was approximately 1.97 ± 0.15 nm, which increased slightly to 2.11 ± 0.20 nm after incorporation of the initiator. Significant differences were observed after the ATRP reaction, with heights reaching 2.46 ± 0.17 nm, but then reduced to 1.95 ± 0.19 nm upon exposure to 365 nm UV light which further confirmed the disassociation of polymer layers from the origami surface owing to the photo‐dynamic change of azobenzene configuration (Figure 2c). The detached polymers were collected and found to be intact (Figure S5). Consequently, we have successfully achieved separation between DNA origami and the patterned polymers without damaging the DNA origami.

**FIGURE 3:**
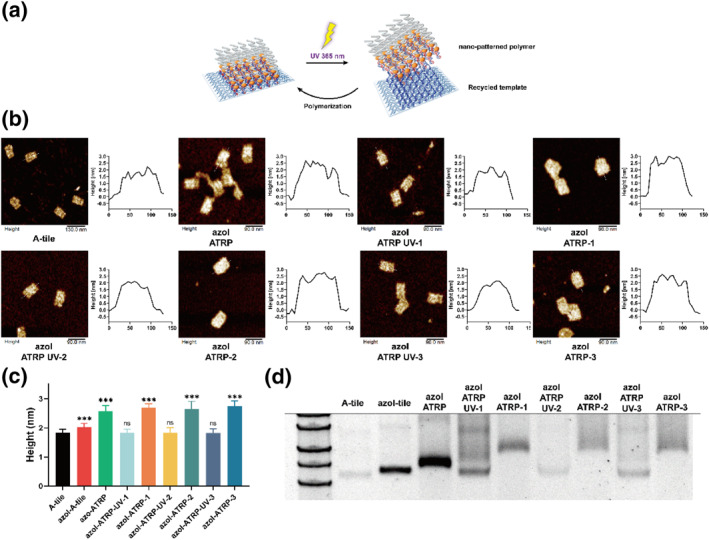
(a) recycle of separated DNA origami template for regeneration of nano‐patterned polymers. (b) AFM images and height profiles of A‐tile, azoI‐ATRP, azoI‐ATRP‐UV‐1, azoI‐ATRP‐1, azoI‐ATRP‐UV‐2, azoI‐ATRP‐2, azoI‐ATRP‐UV‐3, azoI‐ATRP‐3. Scale bars: 90 nm. (c) average height analysis of A‐tile, azoI‐ATRP, azoI‐ATRP‐UV‐1, azoI‐ATRP‐1, azoI‐ATRP‐UV‐2, azoI‐ATRP‐2, azoI‐ATRP‐UV‐3, azoI‐ATRP‐3. Data represent means ± SE, *n* = 30. ***: *p* < 0.005. (d) 2% agarose gel characterization of recycle of polymer layers. Lane 1: A‐tile; lane 2: azoI‐tile; lanes 3: azoI‐ATRP; lanes 4: azoI‐ATRP‐UV‐1; lane 5: azoI‐ATRP‐1; lane 6: azoI‐ATRP‐UV‐2; lane 7: azoI‐ATRP‐2; lane 8: azoI‐ATRP‐UV‐3; lane 9: azoI‐ATRP‐3. AFM, atomic force microscopy; ATRP, atom transfer radical polymerization.

By applying initiator sites with precisely designed patterns, it is possible for the fabrication of polymers in nanoscale shape by this strategy. The polymers were ligated with DNA origami tightly by base pairing, it was hard to obtain those predefined nano‐patterned polymers without damage to DNA origami, usually in a deconstructive way by heating or acid treatment to remove all the DNA shells.[^4^, ^5^, ^6^] Here we have proved we could separate DNA origami template with polymers in a soft way without damage to origami by photoresponsiveness of azobenzene tethered DNA and DNA origami could easily be collected by conventional PEG purification method. Further, in our concept, UV light exposure induced the dissociation of DNA origami from nano‐patterned polymers, leaving the intact DNA origami which still reserved the reaction site, enabling their collection and maximal reuse. We aimed to verify the feasibility of reusing these DNA origami templates cyclically (Figure 3a). After the ATRP reaction, 365 nm UV light was applied to separate origami template, and collection of them to hybridize with newly azoI‐DNA for growth PEGMEMA polymers again for one turn, three rounds were performed to investigate the integrity of origami template for regeneration of polymers. Following the first cycle, the DNA origami was purified using the PEG method before restarting the ATRP reaction using the same protocol and UV treatment. AFM assays were conducted to observe the morphology of the DNA origami throughout the UV cycles (Figure 3b). Similar to previous observations, after the first ATRP‐UV cycle, the DNA origami collected still attained intact structure but the morphology became clearer and edge showed a solid rectangular shape. The second round demonstrated a similar result with the first one. AzoI‐ATRP‐2 maintained its rough morphology, and the denser polymers filled the origami space, while the polymer layers were peeled off after the UV irradiation. This indicated that azoI‐tile template not only retained its intact structure but also its ability to interact with initiator DNA, form the macroinitiator, and initiate the ATRP reaction, also with the third round. As confirmed by AFM observations following the three UV exposure and ATRP. Height analysis corroborated our observations (Figure 3c): bare azoI‐tile measured 1.83 ± 0.12 nm, grew to 2.58 ± 0.19 nm after ATRP, and was reduced back to 1.83 ± 0.13 nm following UV treatment and then increased to 2.69 ± 0.14 nm in the first cycle. In the second cycle, the height of azoI‐ATRP‐UV‐2 dropped back to 1.83 ± 0.18 nm, and peaked to 2.66 ± 0.26 nm. In the third cycle, the height of azoI‐ATRP‐UV‐3 decreased to 1.82 ± 0.14 nm after UV exposure and then after ATRP, increased to 2.74 ± 0.18 nm. Additionally, a 2% agarose gel was run to document the changes throughout the process (Figure 3d). As depicted, the macroinitiator exhibited minor shift from A‐tile. After polymer growth, the increased molecular weight caused azoI‐ATRP to migrate slowly. Following UV exposure, it migrated as quickly as A‐tile, although this ATRP pattern varied in subsequent rounds, returning to a similar pattern by the UV treatment in both three rounds. And the recycling efficiency in each round was consistently kept in 70%–80% which is mainly dependently on the purification efficiency (Figure S6). These results support the notion that DNA origami templates can be flexibly reused by incorporating azobenzene light‐sensitive molecules, without causing damage.

**FIGURE 4:**
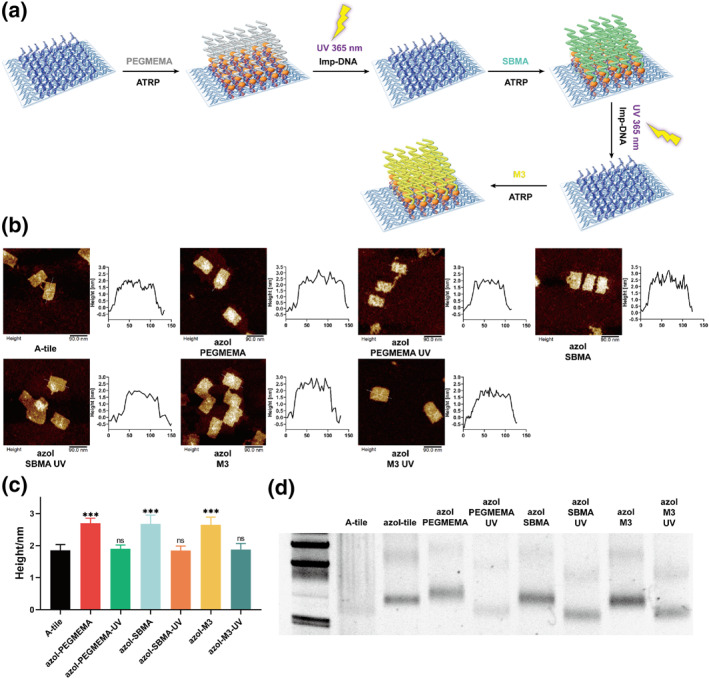
(a) multi‐patterned polymers synthesis by photoresponsive azobenzene modified DNA origami template. (b) AFM images and height profiles of A‐tile, azoI‐PEGMEMA, azoI‐PEGMEMA‐UV, azoI‐SBMA, azoI‐SBMA‐UV, azoI‐M3, azoI‐M3‐UV. Scale bars: 90 nm. (c) average height analysis of A‐tile, azoI‐PEGMEMA, azoI‐PEGMEMA‐UV, azoI‐SBMA, azoI‐SBMA‐UV, azoI‐M3, azoI‐M3‐UV. Data represent means ± SE, *n* = 30. ***: *p* < 0.005. (d) 2% agarose gel characterization of multi‐patterned polymes. Lane 1: A‐tile; lane 2: azoI‐tile; lane 3: azoI‐PEGMEMA; lane 4: azoI‐PEGMEMA‐UV; lane 5: azoI‐SBMA; lane 6: azoI‐SBMA‐UV; lane 7: azoI‐M3; lane 8: azoI‐M3‐UV. AFM, atomic force microscopy; ATRP, atom transfer radical polymerization; DNA, deoxyribonucleic acid.

Next, we explored whether the reused DNA template could be utilized in multi‐polymer synthesis. Three different monomers were sequentially chosen, PEGMEMA, sulfobetaine methacrylate (SBMA), and M3 monomer, the three monomers were added in each round like former mentioned (Figure 4a), after the first reaction, the UV light were immediately applied to separate the origami template and then reuse it into later reaction using second and the third monomers. In the first round, using PEGMEMA as the monomer, the post‐polymerization height was 2.69 ± 0.16 nm, significantly higher than the origami template's 1.85 ± 0.18 nm, indicating successful polymer growth. Following UV exposure, the average height decreased to 1.90 ± 0.12 nm (Figure 4b,c), demonstrating the separation of the origami template from the polymers. In the second round, with SBMA as the monomer, a similar height change was observed post‐ATRP reaction: 2.68 ± 0.27 nm for azoI‐SBMA and 1.84 ± 0.14 nm for the UV‐treated template. Similarly, the third round, utilizing the reused DNA origami template, yielded an average height of 2.65 ± 0.24 nm, which reduced to 1.87 ± 0.19 nm following UV exposure. It should be noted that the height analysis consistently demonstrated a change between polymerization and UV exposure, highlighting the high efficiency of our dissociation method. Additionally, a 2% agarose gel electrophoresis was conducted to further investigate the ATRP process and UV‐irradiated dissociation of the origami template (Figure 4d). As illustrated in the figure, after the first round of polymerization, the azoI‐PEGMEMA band migrated slower than the macroinitiator but returned to the R‐origami position following UV exposure, indicating complete dissociation of the origami template. In the second and third rounds, it was observed that the bands post‐ATRP reaction closely resembled those of the macroinitiators, attributed to the differing charge properties of the monomers which affect migration speeds.

## 3 CONCLUSION

In conclusion, we have presented the precision fabrication of polymer nanostructures via ATRP reaction on photocleavable DNA origami template. This novel approach facilitates both the dynamic and reversible assembly of DNA origami templates as well as their recycling. Azobenzene was initially incorporated into the initiator DNA, successfully catalyzing the in situ ATRP reactions to synthesize nano‐patterned polymers. Under UV light, isomerization of azobenzene reduces the stability of double‐stranded DNA and induces its dissociation. Introduction of impeding DNA allows detached azobenzene‐tethered DNA to bind stably to impeding DNA, thereby preventing reassociation with the DNA origami, facilitating its easy separation and collection via the conventional PEG purification method to yield quantitative DNA templates. Recycled DNA origami maintained reactive sites for reattachment with initiators, thereby enabling the restart of the ATRP reaction and the growth of subsequent polymers. This process, characterized by its rapidity, reversibility, and high efficiency, permitted the reuse of collected DNA origami templates in three ATRP cycles, and facilitated multi‐polymer synthesis using the same recycled template. This integration of photo‐regulator molecules with in situ ATRP technology on DNA origami enabled the creation of nanoscale precision polymer layers and facilitated its convenient, dynamic, and efficient release alongside DNA origami templates, attributing to potential future large‐scale generation of nano‐patterned polymers and cost reduction. Furthermore, the spatial and temporal control of polymer and DNA association and disassociation may offer extensive potential for exploring DNA‐polymer complex interactions with biological tissues under external stimuli and developing new drug delivery systems with light triggers.

## CONFLICT OF INTEREST STATEMENT

There are no conflicts to declare.

## ETHICS STATEMENT

No animal or human experiments were involved in this study.

## Supporting information

Supporting Information S1

## Data Availability

The data that supports the findings of this study are available in the supplementary material of this article.
