# Supplementary material for: Precision fabrication of polymer nanostructures on recyclable DNA template
Source: Smart Mol. 2024 Jun 11;2(2):e20240006. doi: 10.1002/smo.20240006 (PMC12118301; doi:10.1002/smo.20240006)
Supplement: Supplementary file 1 — Supporting Information S1 [file SMO2-2-e20240006-s001.docx]

**Supporting information**

**Precision fabrication of polymer nanostructures on recyclable DNA template**

Zian Lin,^1^ Xuemei Xu,^2^ Yiwei Shi,^1^ Yuzhou Wu^*,1^

1* Hubei Engineering Research Center for Biomaterials and Medical Protective Materials, Hubei Key Laboratory of Bioinorganic Chemistry & Materia Medica, School of Chemistry and Chemical Engineering, Huazhong University of Science and Technology (HUST), 1037 Luoyu Road, Wuhan 430074, China.

2 Key Laboratory for Green Chemical Process of Ministry of Education, Hubei Key Lab of Novel Reaction & Green Chemical Technology, School of Chemical Engineering and Pharmacy, Wuhan Institute of Technology, Wuhan 430205, China.

**Table of contents**

**1 Experimental methods**

- 1. Materials
  2. Fabrication of DNA tile
  3. Synthesis of DNA tile macroinitiator
  4. In-situ ATRP reaction
  5. Photo-regulated DNA origami releasing
  6. DNA origami template cycling experiment
  7. Multi-polymer synthesis using recycled DNA origami template
  8. Atomic force Microscopy (AFM)
  9. Gel electrophoresis characterization
  10. UV/Vis spectra measurements
  11. Synthesis of azoI-DNA

1. **Results**
   1. Photo-regulated isomerization property of azoI-DNA
   2. Stability of origami under the UV exposure
   3. Direct UV irradiation of azoI-ATRP
   4. Effect of Imp-DNA on DNA template releasing
   5. observation of detached patterned polymers
   6. Recycling efficiency of DNA origami
   7. Design of DNA tile
   8. Synthesis of azoI-DNA

Table S1. The list of staple DNA sequences（A-tile）

1. **references**

**1 Experimental methods**

- 1. **Materials**

M13mp18 ssDNA scaffold was obtained from phage transfected Escherichia coli according to the method reported in the literature.^[1]^ "Staple" short strand DNA and DNA short strand were purchased from Wuhan jinkairui Bioengineering Co., Ltd. TPMA, PEGMEMA, PEGDEMA and ascorbic acid were purchased in TCI; M3 monomer was obtained from lab fellow, the structure of M3 monomer was described in the S1.11. Tris and EDTA Disodium Salt were purchased from Sigma-Aldrich. Agarose, 5 × TBE buffer, DNA Marker, 6 × loading buffer were purchased from Sangong biotech (Shanghai) Co., Ltd. D-Threoninol, 4-(phenylazo)benzoic acid, Dicyclohexylcarbodiimide (DCC), 1-Hydroxybenzotriazole (H-OBt), 4-dimethylaminopyridine (DMAP), N,N-Diisopropylethylamine and 4,4'-Dimethoxytrityl chloride were purchased from Aladdin. 2-cyanoethyl N,N-diisopropylchlorophosphoramidite was purchased from Wuhu Huaren Science and Technology Co., Ltd.

- 1. **Fabrication of DNA tile**

L-tile and A-tile were assembled respectively by mixing M13mp18 phage DNA of 7k nt with desired staple strands and modified staple strands in 1 × TAE / Mg^2+^ buffer (20 mM Tris, 1 mM EDTA, and 12 mM MgCl_2_, 10 mM Acetic acid, pH 8.0) and annealing from 65 °C to 20 °C over 2 h, followed by purification with polyethylene glycol (PEG) precipitation method.^[2]^ Briefly, assembled L- or A- tile was treated with 15% PEG8000 (w/v), 5 mM Tris, 1 mM EDTA, and 50 mM NaCl. The solution was mixed well and centrifuged at 12000 g, at room temperature (R.T.) for 25 min. The supernatant was removed and the pellet was dissolved in 1 × TAE / Mg2^+^ buffer.

- 1. **Synthesis of DNA tile macroinitiator**

L-tile or A-tile (0.5 pmol in 50 μL 1 × TAE / Mg2^+^ buffer) were incubated with DNA initiator (0.5 nmol in 5 μL aqueous solution) at room temperature for 4 h, used as L- or A- tile-initiator without any purification. 0.264 μL of 20 × TAE / Mg2^+^ buffer was added to the reaction mixture to keep the constant concentration of Mg^2+^. The excess amount of DNA-initiator serves as sacrificial initiator in the ATRP polymerization. For azoI-DNA synthesis, the conventional initiator was replaced by azoI-DNA, the same procedure as normal DNA tile macroinitiator.

- 1. **In-situ ATRP reaction**

A catalyst stock solution of CuBr_2_ (0.45 mg, 0.002 mmol) and Tris (2-pyridylmethyl) amine (TPMA,4.64 mg, 0.016 mmol) were prepared in 100 μL of N,N-Dimethylformamide (DMF) and ultrapure water. The ascorbic acid stock solution, which can generate the active catalyst species, was prepared at 5 mM in 50 mM NaCl, followed by degassing with argon bubbling for 40 mins. To conduct the polymerization reaction, PEGMEMA (Mn = 300, 2.4 mg, 8 μmol), the DNA origami initiator/azoI-DNA, the catalyst stock solution (1 μL), 20 x TAE buffer (4 μL) were added. Under these conditions, the ratio of the monomer versus the total initiator concentration is 8000 to 1. The reaction solution was degassed with three freeze–pump–thaw cycles and then filled with argon. Ascorbic acid solution (36 μL) was feed into the reactor by a syringe pump at the speed of 0.3 μL/min under stirring. The pump was turned off after 2 h and the reactor was incubated for another 4 h. The mixture after polymerization was purified by 15 % PEG precipitation method ^[2]^ to obtain L-origami-polymer or A-origami-polymer.

- 1. **Photo-regulated DNA origami releasing**

Collecting the mixture after polymerization purified by PEG purification, as for photo-regulated DNA origami template releasing, 100 uL mixture of azoI-ATRP in the tubes were placed and the vial was open for UV exposure, 365 nm UV light was used at a fixed distance in a final intensity of 70-100 mW/cm^2^ from the top of the tubes for 10 min at ambient temperature, after the UV irradiation, imp-DNA ,10 fold excess of initiator was added into the mixture immediately for 10 min, to obtain the released DNA origami template, the mixture was also purified by 15% PEG purification, centrifuged at 12000 g, at room temperature (R.T.) for 25 min, precipitate was collected and dissolved in 1 × TAE / Mg^2+^ buffer.

- 1. **DNA origami template cycling experiment**

After ATRP reaction, for the first round of recycling of DNA origami template, azoI-ATRP mixture was collected and then applied with UV exposure to separate nanopatterned polymers with origami template as in 1.5 depicted. The separated DNA origami template was collected by PEG purification and redissolved in 1 × TAE / Mg^2+^ buffer. The concentration of DNA origami template was quantified by UV absorbance at 260 nm. For the first round of ATRP, quantified DNA origami template, PEGMEMA (Mn = 300, 2.4 mg, 8 μmol), azoI-DNA, the catalyst stock solution (1 μL), 20 x TAE buffer (4 μL) were added. Under these conditions, the ratio of the monomer versus the total initiator concentration is 8000 to 1. The reaction solution was degassed with three freeze–pump–thaw cycles and then filled with argon. Ascorbic acid solution (36 μL) was feed into the reactor by a syringe pump at the speed of 0.3 μL/min under stirring. The pump was turned off after 2 h and the reactor was incubated for another 4 h. the azoI-ATRP-1 was obtained after the reaction. For the second and third round of azoI-ATRP-UV-2, azoI-ATRP-2, azoI-ATRP-UV-3, azoI-ATRP-3 was similar fabricated with the same method.

- 1. **Multi-polymer synthesis using recycled DNA origami template**

DNA origami template, PEGMEMA (Mn = 300, 2.4 mg, 8 μmol), azoI-DNA, the catalyst stock solution (1 μL), 20 x TAE buffer (4 μL) were added. Under these conditions, the ratio of the monomer versus the total initiator concentration is 8000 to 1. The reaction solution was degassed with three freeze–pump–thaw cycles and then filled with argon. Ascorbic acid solution (36 μL) was feed into the reactor by a syringe pump at the speed of 0.3 μL/min under stirring. The pump was turned off after 2 h and the reactor was incubated for another 4 h. azoI-PEGMEMA was obtained as depicted, then UV light in a final intensity of 70-100 mW/cm^2^ was selected for 10 min, the azoI-PEGMEMA-UV was collected by PEG purification and quantified by UV absorbance at 260 nm for next generation for SMBA polymer growth. AzoI-SBMA, azoI-SBMA-UV, azoI-M3, azoI-M3-UV was conducted using the same method.

- 1. **Atomic force Microscopy (AFM)**

Imaging and quantitative nanomechanical property mapping (QNM) were performed with a Bruker Dimension FastScan Bio AFM equipped with the ScanAsyst mode or the QNM imaging mode. The sample solution was deposited onto freshly cleaved mica surface, and left for 5 min at room temperature to allow adsorption of the DNA origami structures. After rinse of 100 μL of 1 × TAE / Mg^2+^ buffer three times, the mica was dried by pure Argon gas. The sample was scanned with the scan rates between 1 and 3 Hz. Several AFM images were acquired at different areas of the mica surface to ensure the reproducibility of the results. All images were analyzed by using the NanoScope Analysis 1.50 software. The height profiles of 3D DNA origami structures were analyzed to calculate the average height and standard error (S.E.).

- 1. **Gel electrophoresis characterization**

For agarose gel electrophoresis, 2% agarose was used for characterization of different assemblies of DNA origami. The gel was run in iced 1×TBE/Mg^2+^ buffer at 90 V for 2 hour. For polyacrylamide gel electrophoresis (PAGE), 12% native-PAGE was used for characterizing the duplex or single-strand oligonucleotides. The gel was run in 1×TBE buffer at 120 V for 1 hour at room temperature in the dark.

- 1. **UV/Vis spectra measurements**

The UV/Vis spectra of azobenzene derivatives tethered on DNA were measured at 25 ℃ and 37 ℃ with UV-Vis spectrophotometer (themofisher). To obtain the trans-form, the solution was kept at 90 ℃ for 3 h. the isomerization property of azoI-DNA were measured under the UV light at different UV intensity (10 mW/cm^2^ and 70-100 mW/cm^2^), the UV spectra was obtained from 300 nm to 500 nm. To investigate the isomerization property of azoI-DNA from cis form to trans form, a white light lamp was used for the irradiation in 20 min and the UV spectra was measured from 300 nm to 500 nm.

- 1. **Synthesis of azoI-DNA**

Compound 1: In a 10 ml round-bottomed flask, a solution of D-threoninol (l00 mg, 0.95 mmol), 4-(phenylazo)benzoic acid (238 mg, 1.05 mmol), dicyclohexylcarbodiimide (DCC) (217 mg, 1.05 mmol) and hydroxybenzotri azole (HOBt) (142 mg, 1.05 mmol) in 5.3 ml dry DMF was stirred under a nitrogen atmosphere at room temperature. The reaction was checked by TLC with 7:3 (vol/vol) ethyl acetate/petroleum ether mobile phase. The reaction mixture was filtered and then a 5-fold volume of water was added, followed by a one-fold volume of diethyl ether to extract the product. The extraction was repeated for a total of five times. The organic phase was washed with saturated NaHCO_3_ solution and saturated NaCl solution and dried over anhydrous MgSO_4_. The solvent was removed with a rotary vacuum evaporator, and the residue was purified by column chromatography (ethyl acetate/methanol: 25/1) and dried to afford compound 1. ^1^H NMR: (400 MHz, CDC1_3_): 7.90-8.00 ppm (m, 6H) , 7.45-7.55 ppm (m, 3H), 7.03-7.08 ppm (d, 1H), 4.29-4.35 ppm (m, 1H), 4.05-4.10 ppm (m, 1H), 3.95-4.00 ppm (d, 2H), 1.25-1.32 ppm (d, 3H).

Compound 2: Compound 1 (100 mg, 0.32 mmol) and 4-dimethylaminopyridine (1.95 mg, 0.016 mmol) were dissolved in 1.80 mL dry pyridine in a 10 mL round-bottomed flask under dry nitrogen. In a separate 10 mL round-bottomed flask, 4,4’-dimethoxytrityl chloride (338.83 mg, 0.384 mmol) was dissolved in 0.5 ml dry CH_2_Cl_2_ under nitrogen, and this solution was added to the above pyridine solution slowly under dry nitrogen on an ice bath with stirring. The mixture was stirred for Ih at 0 ℃ and then at room temperature for another 24h. The reaction was checked by TLC with 30:70:3 (vol/vol/vol) ethyl acetate/petroleum ether/triethylamine. The solvent (pyridine and CH_2_Cl_2_) was removed using a rotary evaporator, and the residue was purified by column chromatography (eluent: 30:70:3 (vol/vol/vol) ethyl acetate/petroleum ether/triethylamine) and dried to afford compound 2 as an orange-red solid. ^1^H NMR (400 MHz, CDC1_3_)： 7.95-6.85 ppm (m,23H), 4.20-4.30 ppm (m, 1H), 4.10-4.20 ppm (m, 1H), 3.65-3.80 ppm (s, 6H), 3.57-3.63, 3.38-3.43 ppm (dd, 2H), 1.20-1.30 ppm (d, 3H).

Compound 3: To a solution containing compound 2 (100 mg, 0.16 mmol) in anhydrous CH_2_CI_2_ (0.59 mL) at 0 ℃, N, N'-diisopropylethylamine (DIPEA) (56.9 mg, 30.0 mmol) was added slowly under nitrogen. Then, 2-cyanoethyl diisopropyl chlorophosphoramidite (42 μL, 13 mmol) was added dropwise, and the reaction mixture was stirred at 0 ℃ for 1 h. The reaction was checked by TLC with 40:60:3 (vol/vol/vol) ethyl acetate/petroleum ether/triethylamine. After removing the solvent, the residue was dissolved in ethyl acetate, and the organic phase was washed with saturated NaHCO_3_ solution and NaCl solution and dried over anhydrous MgSO_4_. The solvent was evaporated below 30 ℃, and the residue was purified by column chromatography and dried to afford compound 3. 'H NMR (400 MHz, CDCl_3_): 8.00-6.79 ppm (m, 22H), 6.62 ppm (d, 1H), 4.48 ppm (m, 1H), 4.39 ppm (m, 1H), 4.21-4.10 ppm (m, 2H), 3.76-3.77 ppm (s, 6H), 3.57-3.34 ppm (m, 4H), 2.76-2.72 ppm (m, 2H), 1.30-1.25 ppm (m, 15H). ^31^P (CDCI_3_): 149.


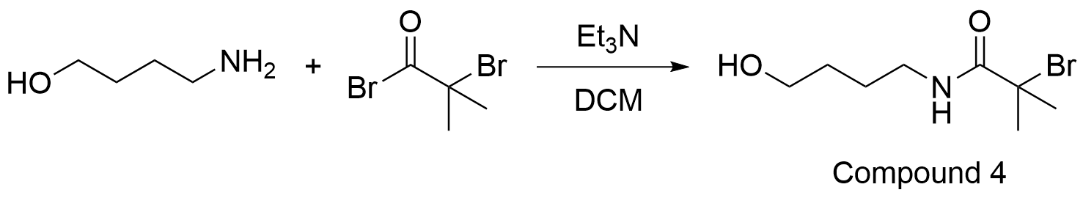


Compound 4: The 4-Amino-1-butanol (1 g, 11.22 mmol) and triethylamine (1.25 g, 12.48 mmol) were dissolved in 10 mL of dichloromethane and α-bromoisobutyryl bromide (2.875 g, 11.96 mmol) was added drop wise. The reaction was stirred for 16 hours. The reaction mixture was filtered and stirred with 10 ml of 5% KOH for 2 hours. The reaction mixture was then added to a separatory funnel and the aqueous layer was separated. The organic layer was then washed with 1N NaOH (10 ml, 2X), 1N HCl (10 ml, 2X) brine (10 ml, 1X) dried over Na_2_SO_4_ filtered and the solvent was evaporated. ^1^H NMR (300 MHz, CDCl_3_): 7.0 ppm (s, 1H) 3.7 ppm (t, 2H) 3.3 ppm (t, 2H), 2.2 ppm (s, 1H), 1.9 ppm (s, 6H), 1.6 ppm (m, 4H).


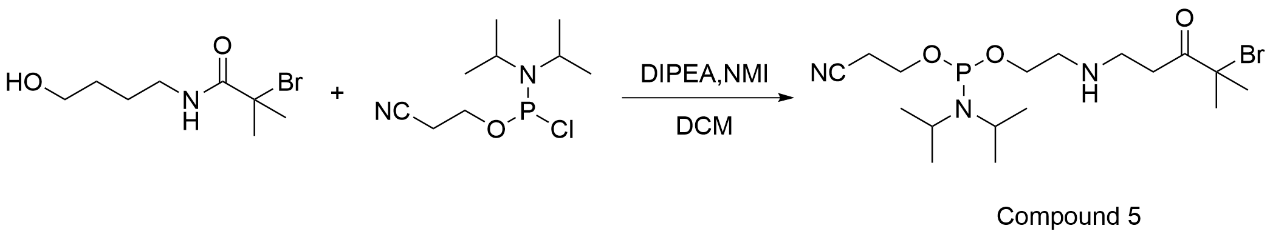


Compound 5: To a solution of 1 (300 mg, 1.27 mmol) in CH_2_Cl_2_ (10 mL), DIPEA (1.1 mL, 6.32 mmol), 2-cyanoethyl-N,N-diisopropyl-chloro-phosphoramidite (424 µL, 1.89 mmol) and 1-methyl-imidazole (57 µL, 0.713mmol) were added. The mixture was stirred for 30 mins at 0 °C and 1.5 hour at R.T. Work up was done with NaHCO_3_ (saturated)/EtOAc. Column chromatography (EtOAc/Hexane/Triethylamine, 1:3:0.5) gives the product (360 mg) in 76.5 % isolated yield. ^1^H NMR (400 MHz, CDCl_3_) δ 6.78 (s, 1H), 3.91-3.56 (m, 7H), 3.33-3.28 (m, 2H), 2.64 (t, J = 6.4 Hz, 2H), 1.95 (s, 6H), 1.68-1.63 (m, 4H), 1.18 (dd, J = 6.8, 3.6 Hz, 12H).


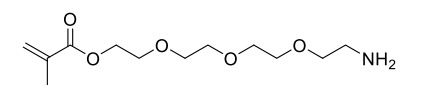


M3 monomer: 1H NMR (400 MHz, CDCl_3_): δ 7.58 (s, 2H), 6.12 (s, 1H), 5.62 (s, 1H), 4.39 – 4.31 (m, 2H), 3.80 (t, J =5.0 Hz, 2H), 3.74 – 3.70 (m, 4H), 3.65 (s, 6H), 3.26 (q, J = 5.9, 5.3 Hz, 2H), 1.93 (s, 3H).

**Synthesis of azoI-DNA by solid DNA synthesizer**

Azobenzene and initiator modified oligonucleotides were synthesized on a 12-Column DNA Synthesizer (PolyGen GmbH) based on the synthesis protocol provided by the reagents’ manufacturers.^1^ Briefly, azobenzene and initiator groups were incorporated into DNA using the phosphoramidite monomer. After machine synthesis, all the DNA products were cleaved from the solid support by incubating with ammonia and methylamine (1:1, v/v) at 65 ℃ for 30 min in a water bath. In order to precipitate DNA, 40 µL 3.0 M NaCl and 1.2 mL ethanol were added and incubated at -20 ℃ for 30 min. After removing the supernatant, the precipitated DNA product was purified by 12% PAGE gel, the DNA product was separated by the PAGE gel and then extracted by cutting the gel into small pieces and sank in the pure water for 48h with shaking. The azoI-DNA was characterized by PAGE gel and MALDI-TOF. The purified strand was dissolved in ultrapure water and quantified by UV absorption at 260 nm.

1. **Results**
   1. **Photo-regulated isomerization property of azoI-DNA**


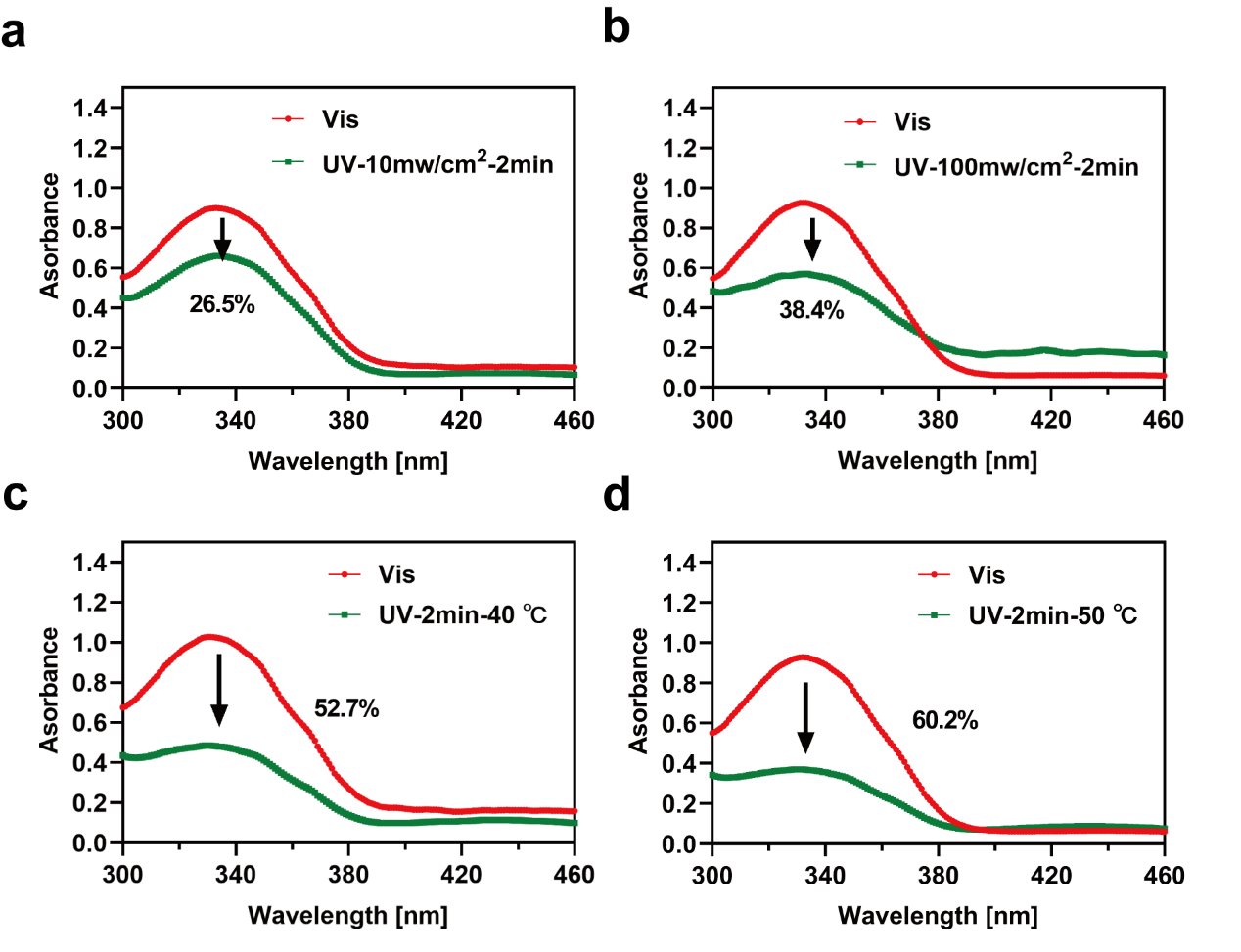


**Figure S1.** Photo-isomerization property of azoI-DNA under various conditions, the azoI-DNA was applied to different UV light intensity of a) 10 mW/cm^2^ and b) 100 mW/cm^2^, and different temperature at c) 40 ℃ and d) 50 ℃. Higher light intensity and higher temperature will accelerate the isomerization speed and increase the efficiency for azobenzene.

- 1. **Stability of origami under the UV exposure**


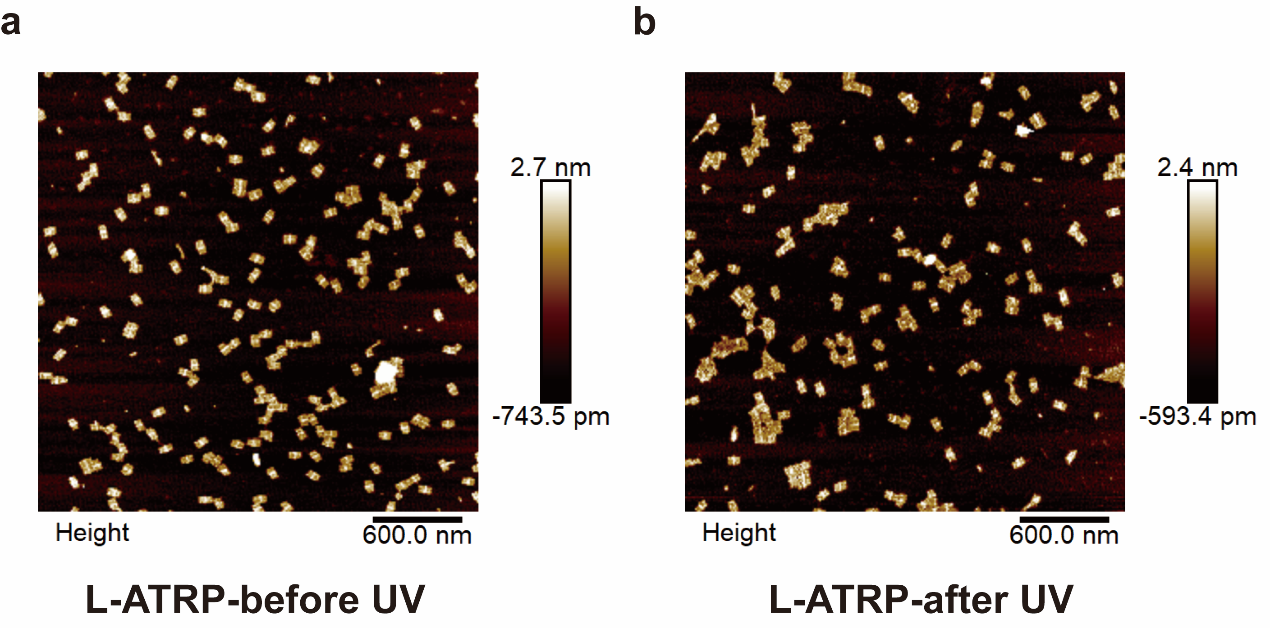


**Figure S2.** AFM characterization of stability of DNA origami before and after UV irradiation, L-origami and ATRP one was chosen, a) before UV exposure, it showed stable and clear rectangular shape of origami and b) after UV light, there is no obvious difference for AFM image, indicate moderate UV light exposure is harmless to DNA origami.

- 1. **Direct UV irradiation of azoI-ATRP**


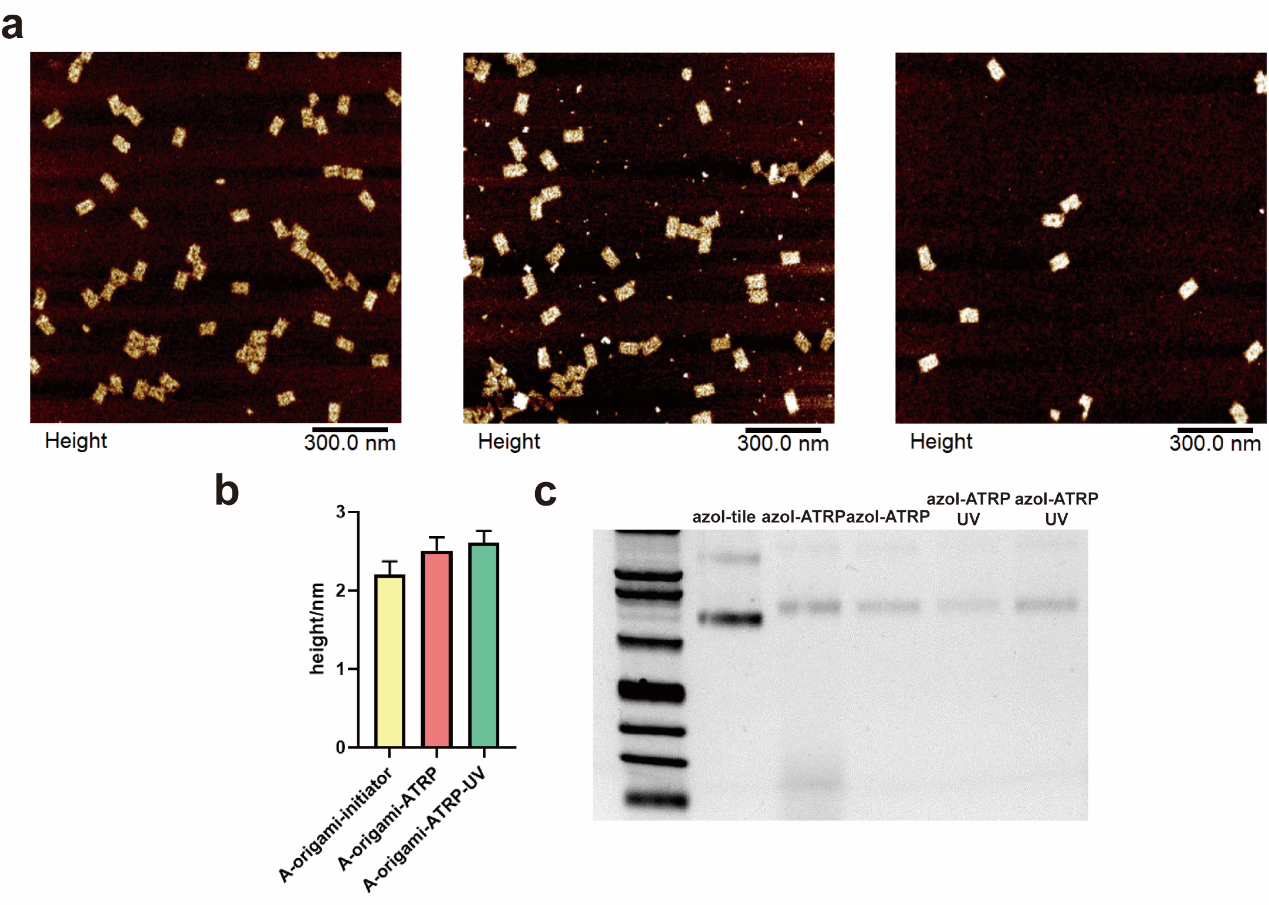


**Figure S3.** UV irradiation of azoI-ATRP, no difference was observed from a) AFM image and b) heigh profile analysis with c) 2% agarose gel, indicate fast reassociation of DNA origami with patterned polymers

- 1. **effect of Imp-DNA on DNA template releasing**


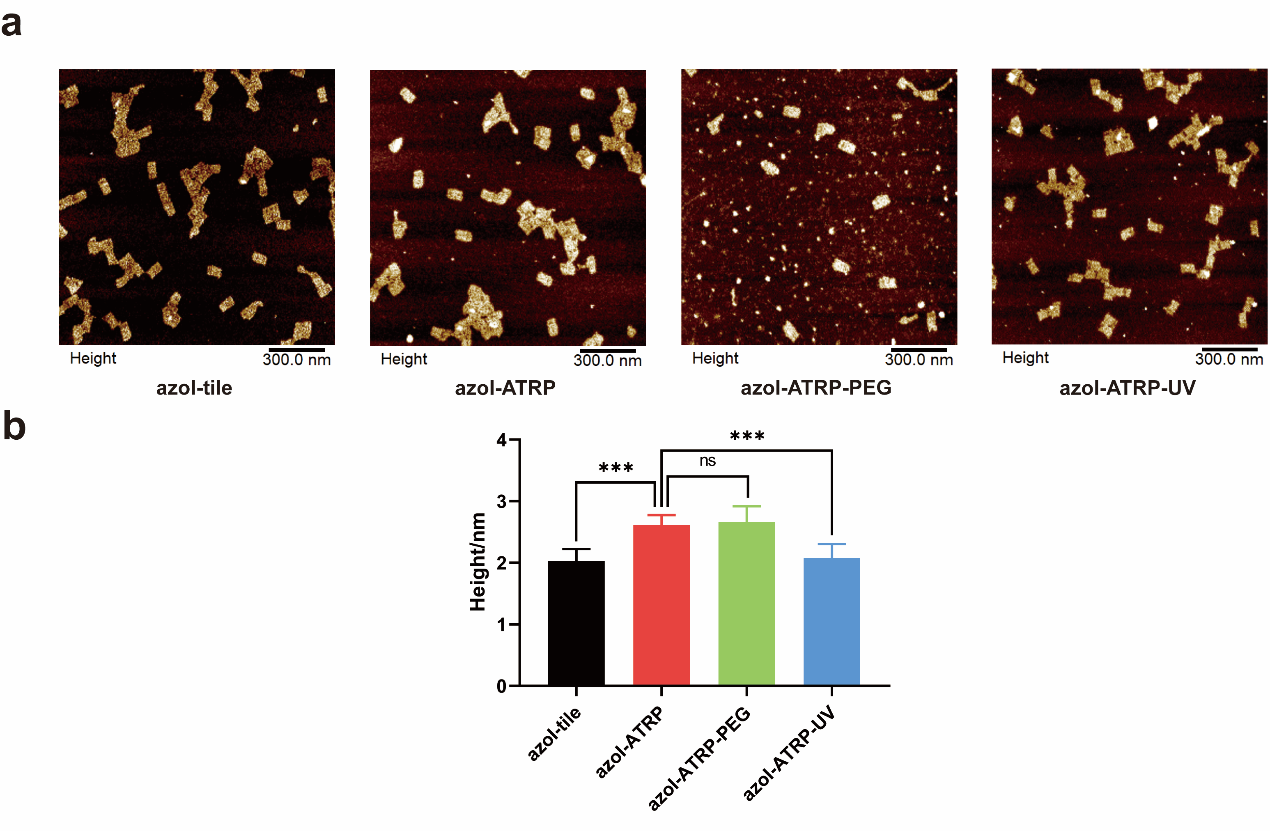


**Figure S4.** Characterization of imp-DNA to investigate if imp-DNA could compete with DNA origami template. AFM images and height profile showed after adding the imp-DNA without UV light (azoI-ATRP-PEG: adding amounts of imp-DNA with azoI-ATRP for 20min without UV irradiation and then purified by PEG purification method), no significant difference was found indicate that excess of imp-DNA will not compete DNA origami template with azoI-DNA even though the concentration was 10 times than azoI-DNA, and only after UV irradiation the image and height profile could be found changed.

- 1. **observation of detached patterned polymers**

**
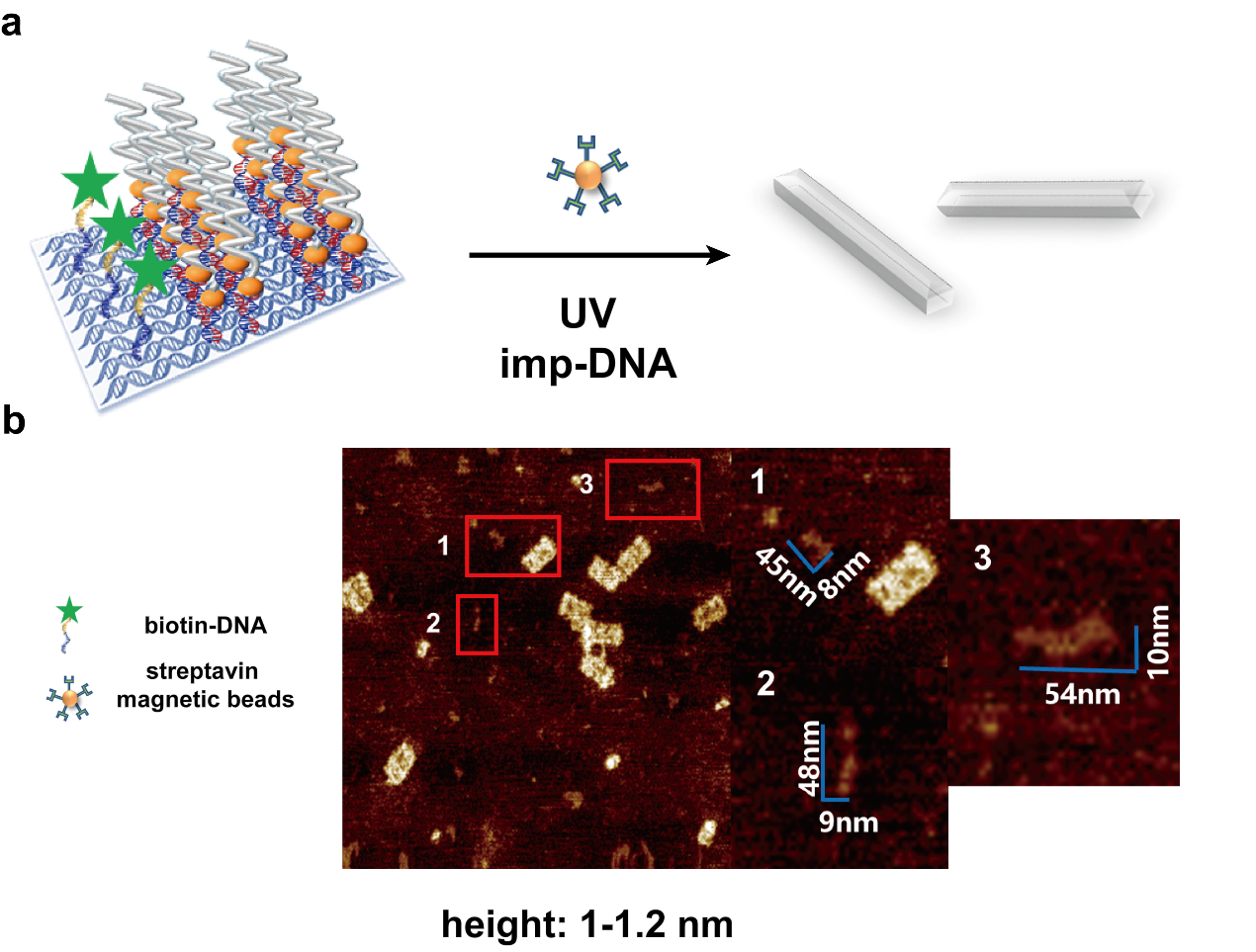
**

**Figure S5.** Characterization of detached patterned polymers. a) the 2L-DNA tile, two lines on each side was chosen, DNA origami was modified with biotin-DNA and could interact with streptavidin tethered magnetic beads. After the polymerization, and exposed under the UV, the DNA origami and patterned polymers could be easily separated by magnet, and the solution left was polymers. b) the separation efficiency of this method is much lower than the PEG purification but without the interference of PEG polymers. we found there were polymers on the mica observed by AFM, which the size of those polymers was 49 * 9 *1 nm^3^, corresponding well with the predesigned pattern.

- 1. **Recycling efficiency of DNA origami**

**
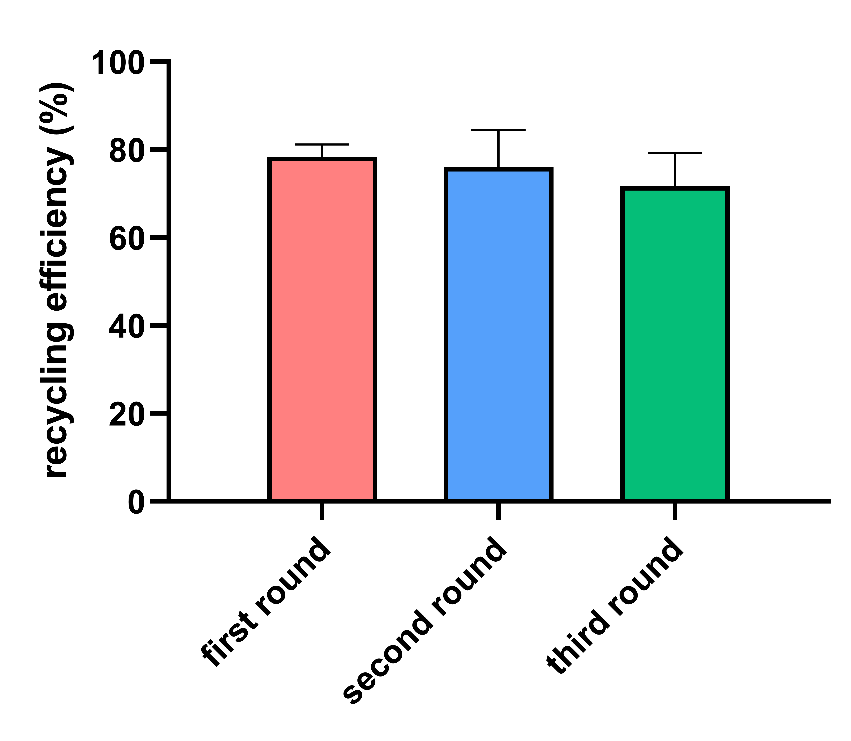
**

**Figure S6.** Recycling efficiency of DNA origami template in each round. The DNA origami template was collected by PEG purification method, and the concentration of DNA origami was read and quantified by UV absorbance at 260 nm. at each round, the recycling efficiency was consistent and kept in 70-80%.

- 1. **Design of DNA tile**


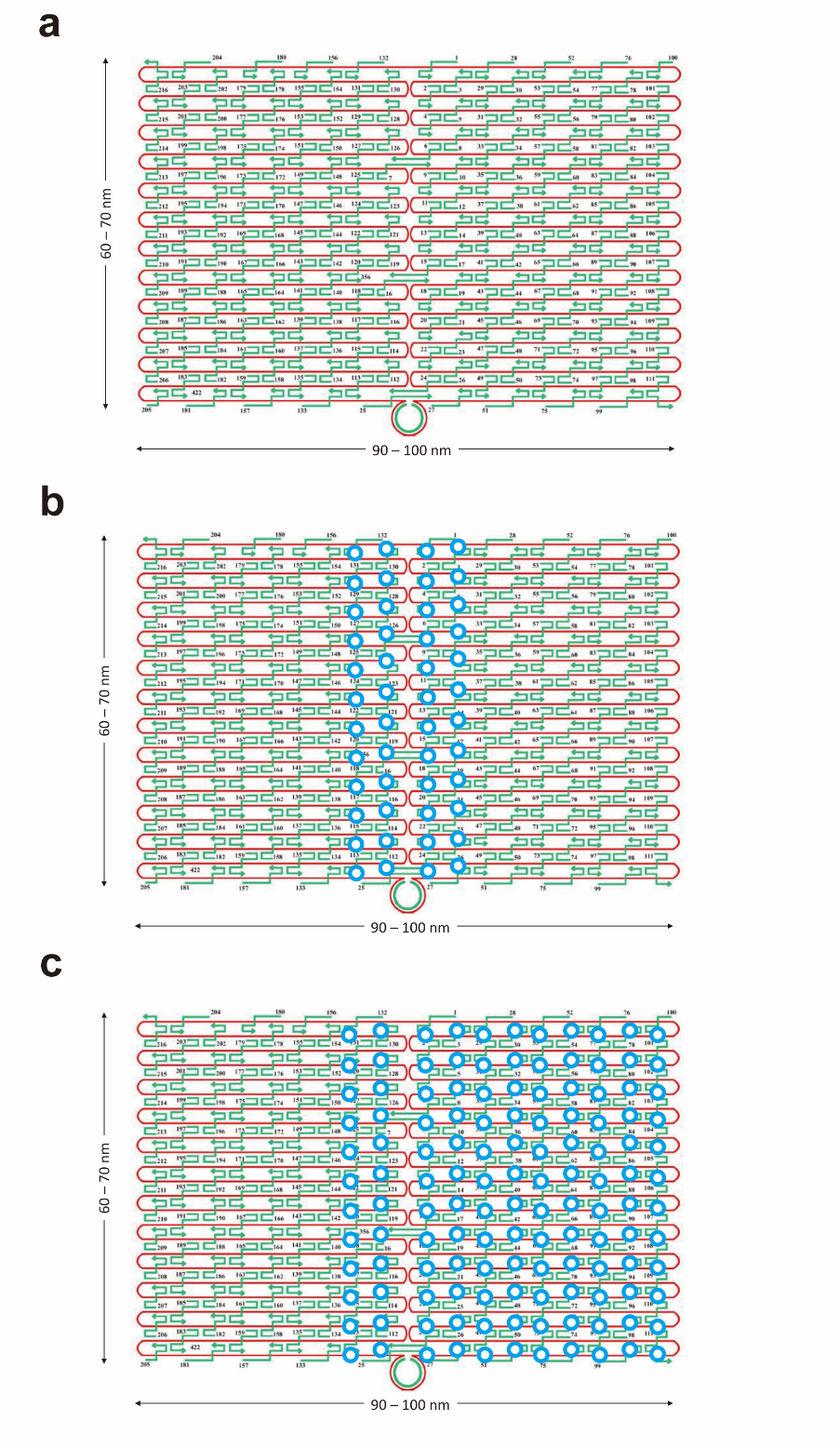


**Figure S6.** Design of DNA nanotile.^[3]^ a) Schematic of DNA tile. b) Schematic of L-tile. c) Schematic of A-tile. The blue circles at figures indicate the positions of linking DNA-initiator staples.

- 1. **Synthesis of azoI-DNA**


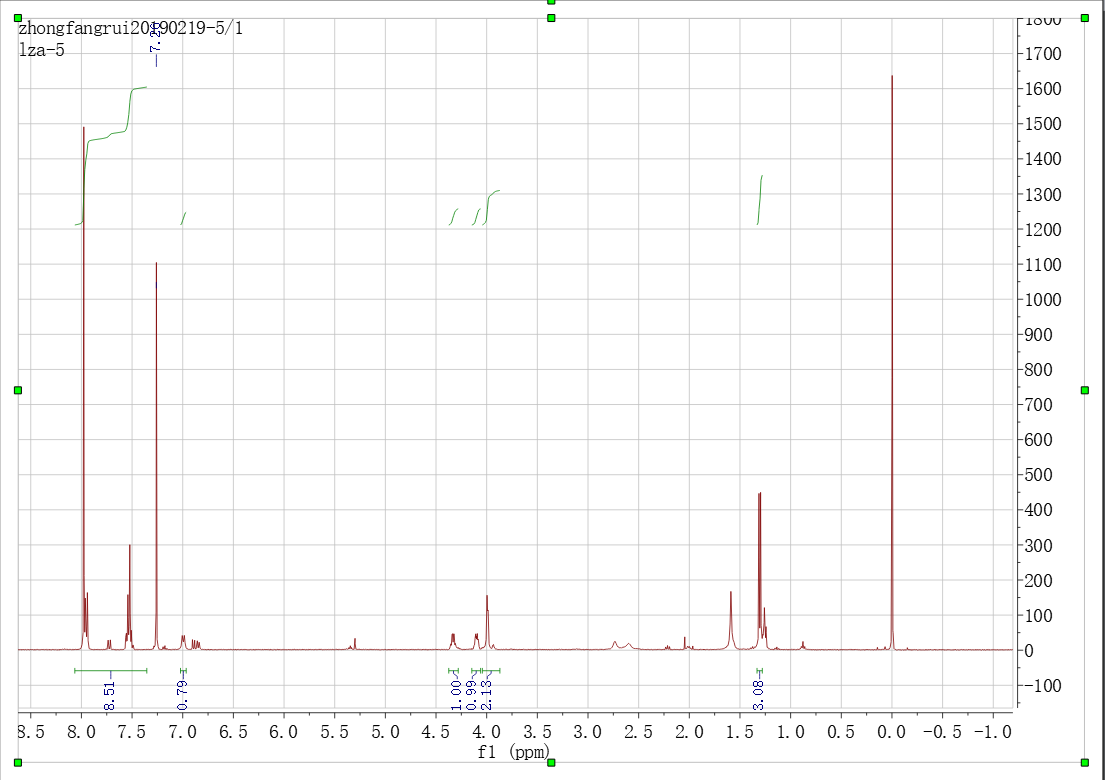


**Figure S7.** ^1^H NMR spectrum of the compound 1: ^1^H NMR (400 MHz, CDCl_3_); δ = 7.96-7.38 (m, 9H, ArH), 7.12 (d, 1H, -NHCO), 4.33 (m, 1H, -CH(OH)CH_3_), 4.09 (m, 1H, HOCH_2_CH(NHCO-)-), 3.98 (d, 2H, -CH2-OH), 1.29 (d, 3H, -CH(OH)CH_3_).


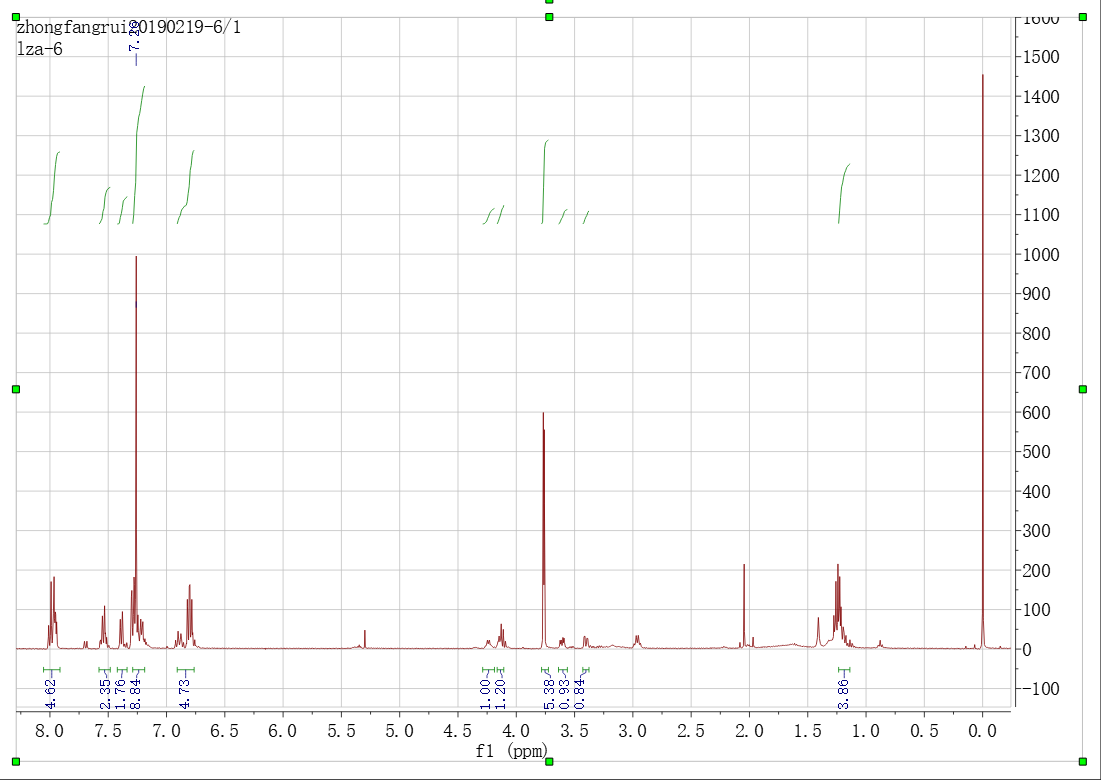


**Figure S8.** ^1^H NMR spectrum of the compound 2: ^1^H NMR (400 MHz, CDCl_3_); δ= 8.00-6.78 (m, 23H, ArH of DMT, azobenzene and -NHCO-), 4.25 (m, 1H, -CH(OH)CH_3_),4.17 (m, 1H, -OCH_2_CH(NHCO-)-), 3.77 and 3.76 (s, 6H, -C_6_H_4_-OCH_3_), 3.60 and 3.42 (dd, 2H, -CH_2_-ODMT),1.23 (d, 3H, -CH(OH)CH_3_).


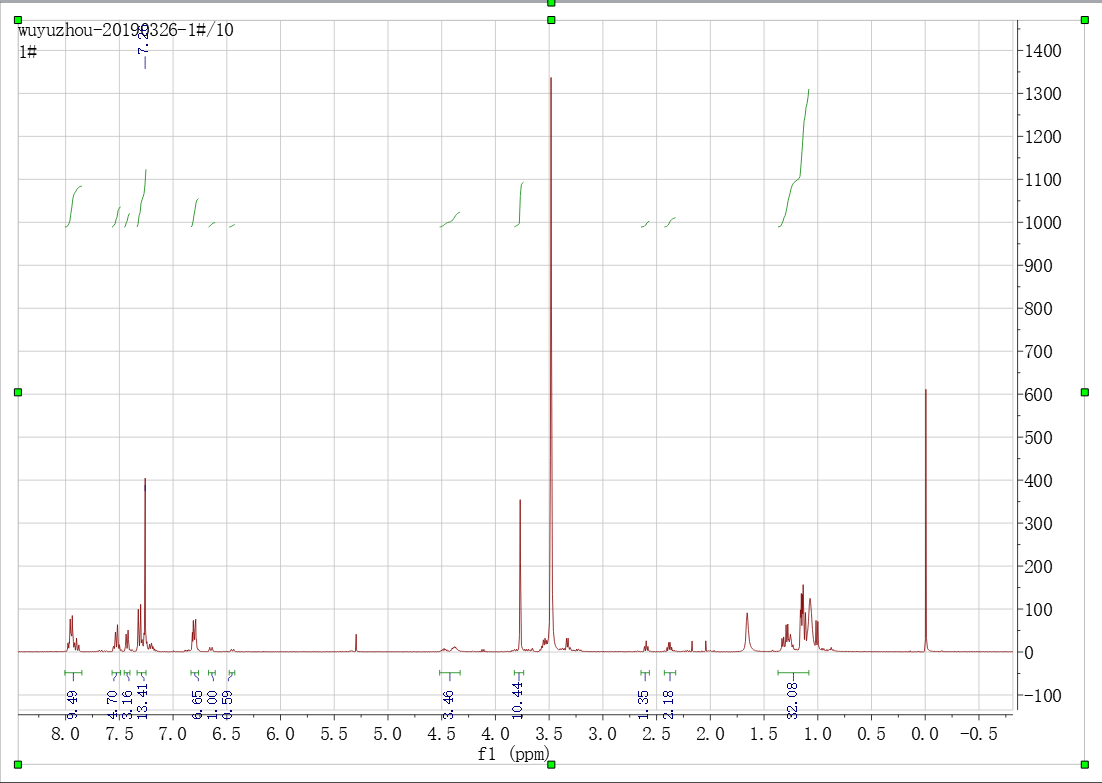


**Figure S9.** ^1^H NMR spectrum of the compound 3: ^1^H NMR (400 MHz, CDCl_3_); δ= 8.00-6.78 (m, 23H, ArH of DMT, azobenzene and -NHCO-), 4.25 (m, 1H, -CH(OH)CH_3_), 4.17 (m, 1H, -OCH_2_CH(NHCO-)-), 3.77 and 3.76 (s, 6H, -C_6_H_4_-OCH_3_), 3.60 and 3.42 (dd, 2H, -CH_2_-ODMT), 1.23 (d, 3H, -CH(OH)CH_3_).


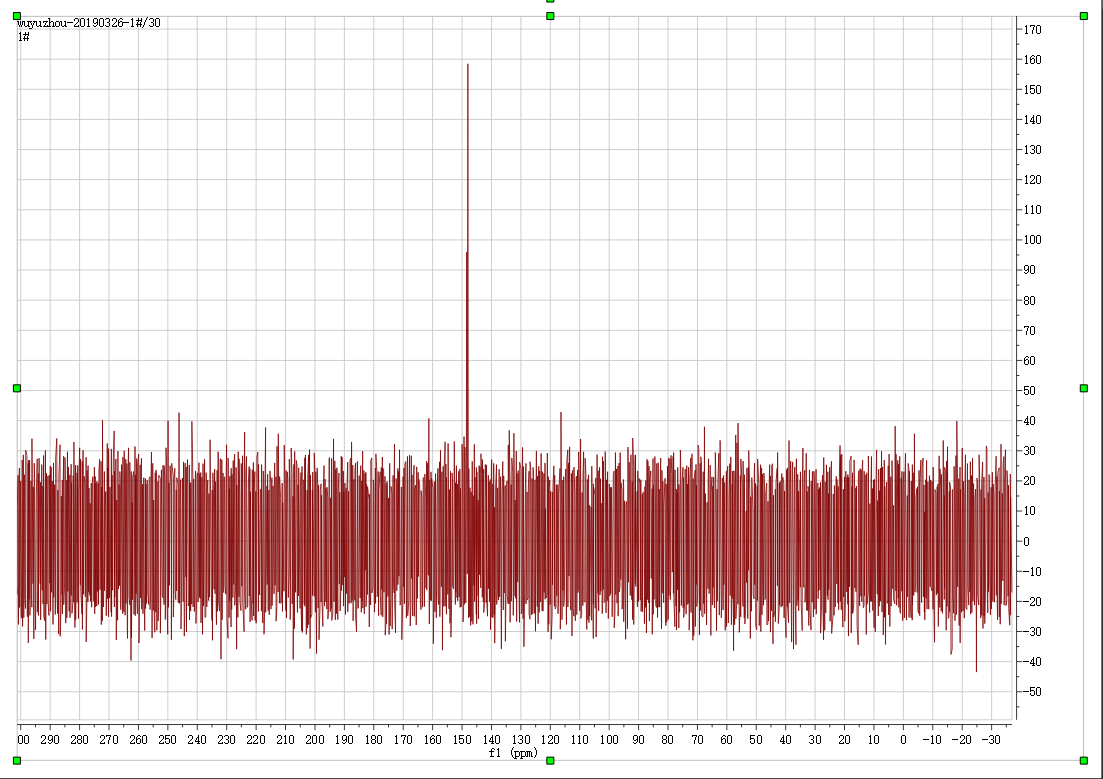


**Figure S10.** ^31^P NMR spectrum of the compound 3: ^31^P NMR (400 MHz, CDCl_3_); δ=149


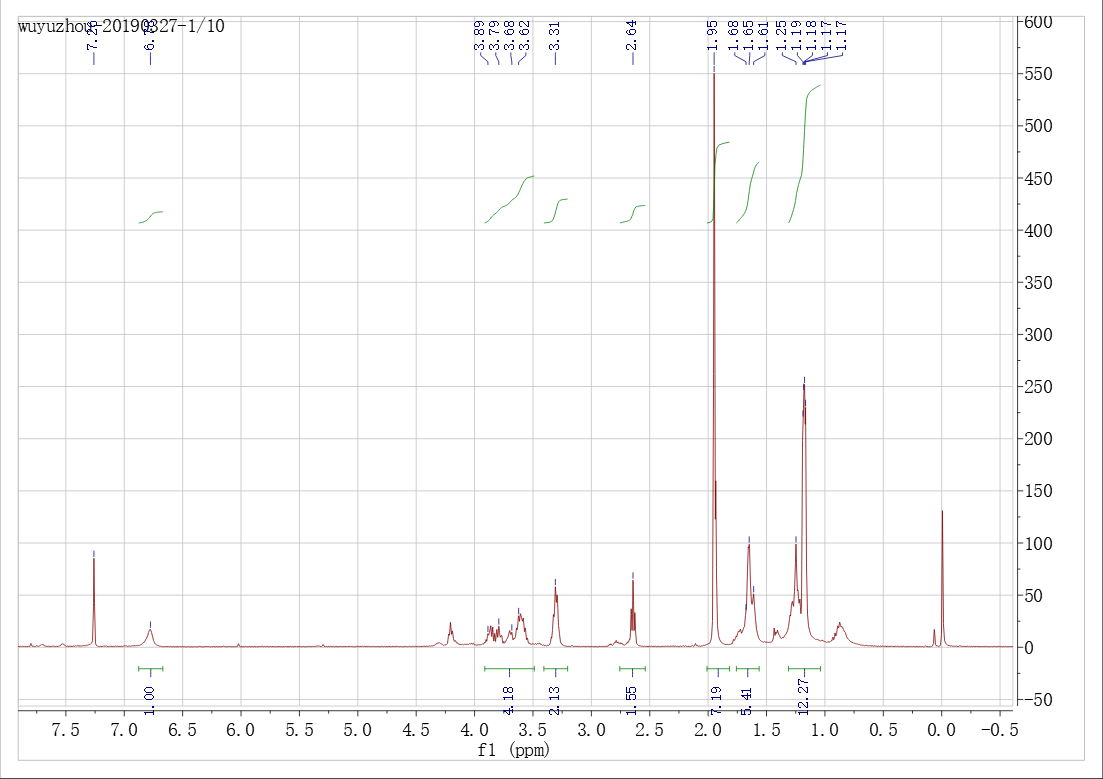


**Figure S11.** ^1^H NMR spectrum of the compound 5: ^1^H NMR (300 MHz, CDCl_3_): δ 1.15 (d, J = 2.6 Hz, 6H), 1.17 (d, J = 2.6 Hz, 6H), 1.60-1.69 (m, 4H), 1.93 (s, 6H), 2.63 (app t, J = 6.44 Hz, 2H), 3.26-3.32 (m, 2H), 3.52-3.91 (m, 6H), 6.77 (s, 1H).


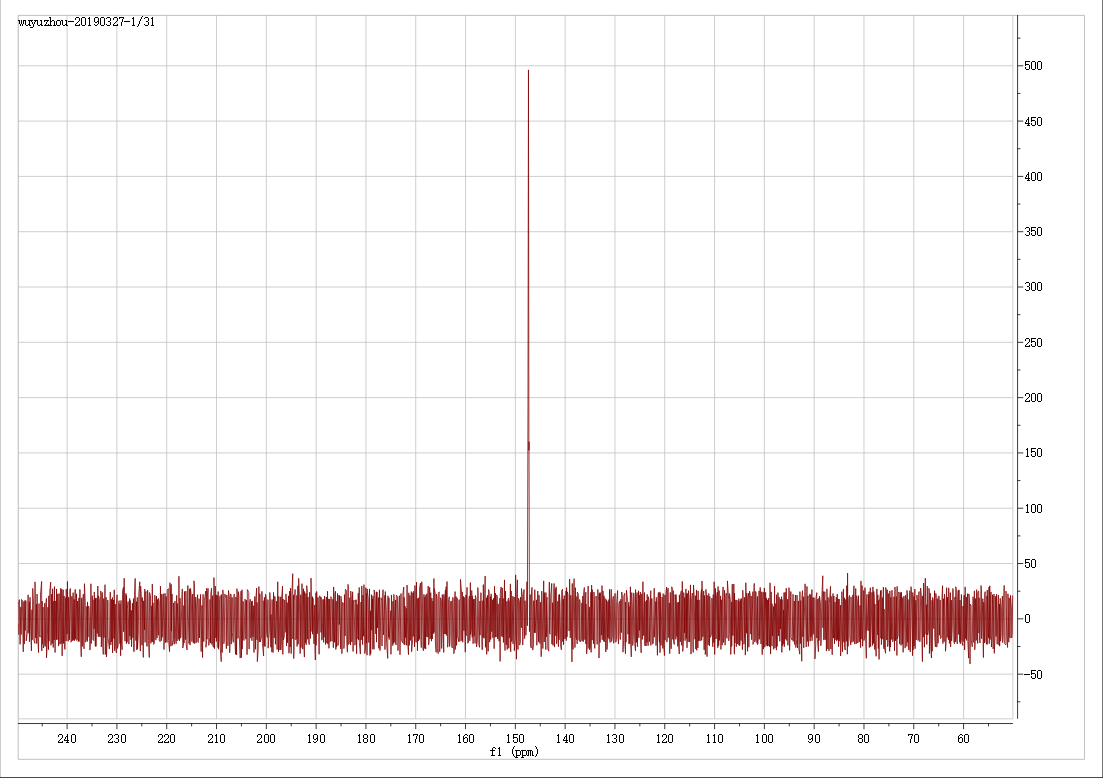


**Figure S12.** ^31^P NMR spectrum of the compound 5: ^31^P NMR (127 MHz, CDCl_3_): 147.65


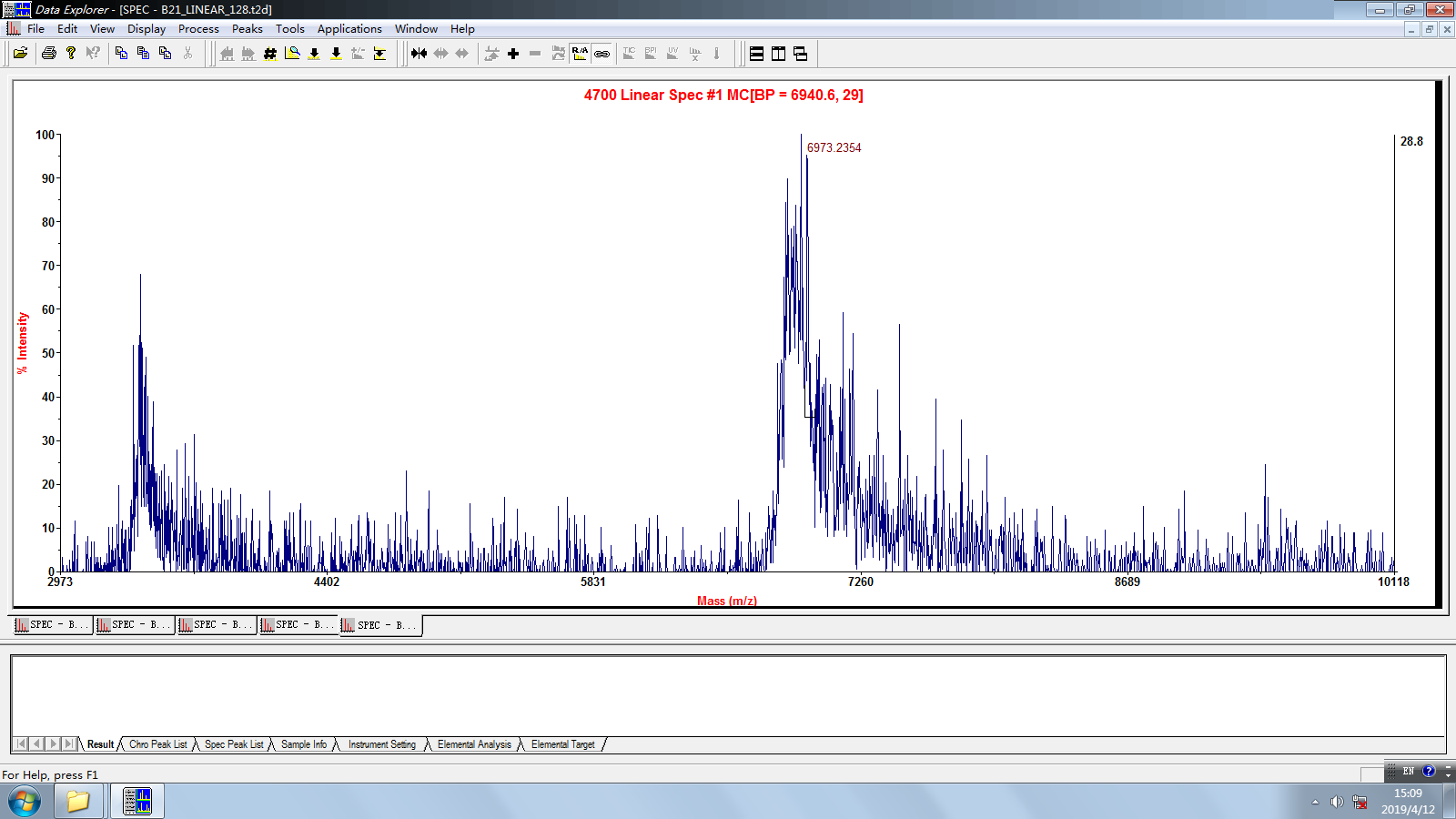


**Figure S13.** Mass spectrum of the azoI-DNA. AzoI-DNA: 5’ initiatorCTCXTAXCCXACXCTXACXTA-3’.


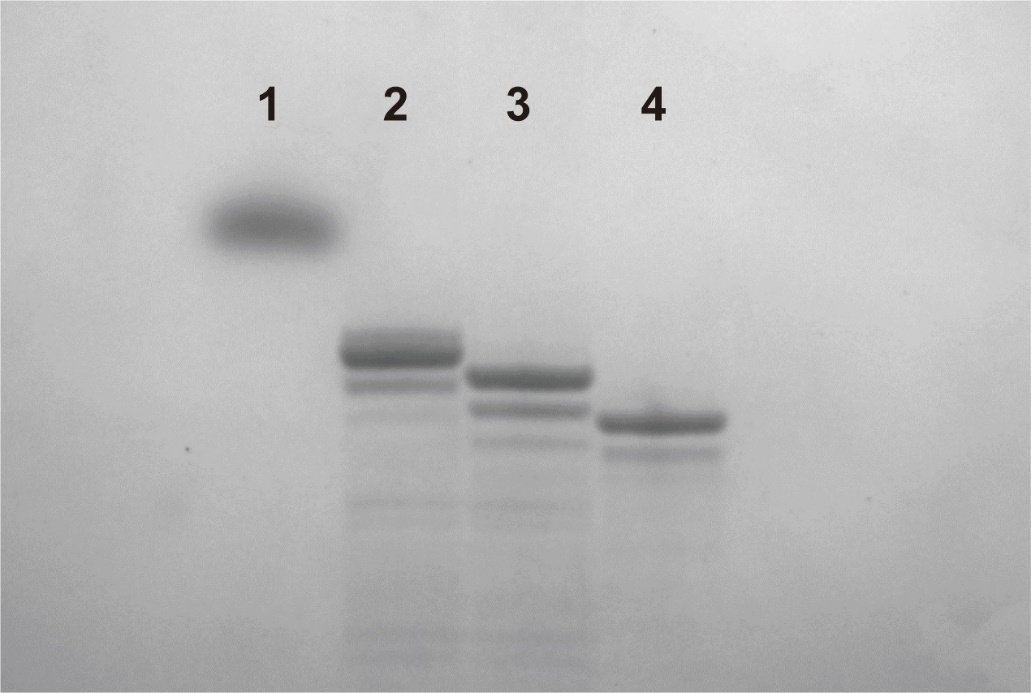


**Figure S14.** Characterization of azoI-DNA by 12% PAGE gel: 1) loading buffer 2) azoI-DNA 3) azoI-DNA without incorporation of initiator 4) control DNA 20nt.

**Table S1. The list of staple DNA sequences （A-tile）**

| **NO.** | **Sequence (5’-3’) xxxxx represents sticky sequence** |
| --- | --- |
| **1** | CAAGCCCAATAGGAACCCATGTACAAACAG  TTTTTTTTAGTAGGTGGTAGAG |
| **2** | AATGCCCCGTAACAGTGCCCGTATCTCCCTCA  TTTTTTTTAGTAGGTGGTAGAG |
| **3** | TGCCTTGACTGCCTATTTCGGAACAGGGATAG  TTTTTTTTAGTAGGTGGTAGAG |
| **4** | GAGCCGCCCCACCACCGGAACCGCGACGGAAA  TTTTTTTTAGTAGGTGGTAGAG |
| **5** | AACCAGAGACCCTCAGAACCGCCAGGGGTCAG  TTTTTTTTAGTAGGTGGTAGAG |
| **6** | TTATTCATAGGGAAGGTAAATATTCATTCAGT  TTTTTTTTAGTAGGTGGTAGAG |
| **7** | CATAACCCGAGGCATAGTAAGAGCTTTTTAAG  TTTTTTTTAGTAGGTGGTAGAG |
| **8** | ATTGAGGGTAAAGGTGAATTATCAATCACCGG  TTTTTTTTAGTAGGTGGTAGAG |
| **9** | AAAAGTAATATCTTACCGAAGCCCTTCCAGAG  TTTTTTTTAGTAGGTGGTAGAG |
| **10** | GCAATAGCGCAGATAGCCGAACAATTCAACCG  TTTTTTTTAGTAGGTGGTAGAG |
| **11** | CCTAATTTACGCTAACGAGCGTCTAATCAATA  TTTTTTTTAGTAGGTGGTAGAG |
| **12** | TCTTACCAGCCAGTTACAAAATAAATGAAATA  TTTTTTTTAGTAGGTGGTAGAG |
| **13** | ATCGGCTGCGAGCATGTAGAAACCTATCATAT  TTTTTTTTAGTAGGTGGTAGAG |
| **14** | CTAATTTATCTTTCCTTATCATTCATCCTGAA  TTTTTTTTAGTAGGTGGTAGAG |
| **15** | GCGTTATAGAAAAAGCCTGTTTAGAAGGCCGG  TTTTTTTTAGTAGGTGGTAGAG |
| **16** | GCTCATTTTCGCATTAAATTTTTGAGCTTAGA  TTTTTTTTAGTAGGTGGTAGAG |
| **17** | AATTACTACAAATTCTTACCAGTAATCCCATC  TTTTTTTTAGTAGGTGGTAGAG |
| **18** | TTAAGACGTTGAAAACATAGCGATAACAGTAC  TTTTTTTTAGTAGGTGGTAGAG |
| **19** | TAGAATCCCTGAGAAGAGTCAATAGGAATCAT  TTTTTTTTAGTAGGTGGTAGAG |
| **20** | CTTTTACACAGATGAATATACAGTAAACAATT  TTTTTTTTAGTAGGTGGTAGAG |
| **21** | TTTAACGTTCGGGAGAAACAATAATTTTCCCT  TTTTTTTTAGTAGGTGGTAGAG |
| **22** | CGACAACTAAGTATTAGACTTTACAATACCGA  TTTTTTTTAGTAGGTGGTAGAG |
| **23** | GGATTTAGCGTATTAAATCCTTTGTTTTCAGG  TTTTTTTTAGTAGGTGGTAGAG |
| **24** | ACGAACCAAAACATCGCCATTAAATGGTGGTT  TTTTTTTTAGTAGGTGGTAGAG |
| **25** | GAACGTGGCGAGAAAGGAAGGGAACAAACTAT  TTTTTTTTAGTAGGTGGTAGAG |
| **26** | TAGCCCTACCAGCAGAAGATAAAAACATTTGA  TTTTTTTTAGTAGGTGGTAGAG |
| **27** | CGGCCTTGCTGGTAATATCCAGAACGAACTGA  TTTTTTTTAGTAGGTGGTAGAG |
| **28** | CTCAGAGCCACCACCCTCATTTTCCTATTATT  TTTTTTTTAGTAGGTGGTAGAG |
| **29** | CTGAAACAGGTAATAAGTTTTAACCCCTCAGA  TTTTTTTTAGTAGGTGGTAGAG |
| **30** | AGTGTACTTGAAAGTATTAAGAGGCCGCCACC  TTTTTTTTAGTAGGTGGTAGAG |
| **31** | GCCACCACTCTTTTCATAATCAAACCGTCACC  TTTTTTTTAGTAGGTGGTAGAG |
| **32** | GTTTGCCACCTCAGAGCCGCCACCGATACAGG  TTTTTTTTAGTAGGTGGTAGAG |
| **33** | GACTTGAGAGACAAAAGGGCGACAAGTTACCA  TTTTTTTTAGTAGGTGGTAGAG |
| **34** | AGCGCCAACCATTTGGGAATTAGATTATTAGC  TTTTTTTTAGTAGGTGGTAGAG |
| **35** | GAAGGAAAATAAGAGCAAGAAACAACAGCCAT  TTTTTTTTAGTAGGTGGTAGAG |
| **36** | GCCCAATACCGAGGAAACGCAATAGGTTTACC  TTTTTTTTAGTAGGTGGTAGAG |
| **37** | ATTATTTAACCCAGCTACAATTTTCAAGAACG  TTTTTTTTAGTAGGTGGTAGAG |
| **38** | TATTTTGCTCCCAATCCAAATAAGTGAGTTAA  TTTTTTTTAGTAGGTGGTAGAG |
| **39** | GGTATTAAGAACAAGAAAAATAATTAAAGCCA  TTTTTTTTAGTAGGTGGTAGAG |
| **40** | TAAGTCCTACCAAGTACCGCACTCTTAGTTGC  TTTTTTTTAGTAGGTGGTAGAG |
| **41** | ACGCTCAAAATAAGAATAAACACCGTGAATTT  TTTTTTTTAGTAGGTGGTAGAG |
| **42** | AGGCGTTACAGTAGGGCTTAATTGACAATAGA  TTTTTTTTAGTAGGTGGTAGAG |
| **43** | ATCAAAATCGTCGCTATTAATTAACGGATTCG  TTTTTTTTAGTAGGTGGTAGAG |

| **44** | CTGTAAATCATAGGTCTGAGAGACGATAAATA  TTTTTTTTAGTAGGTGGTAGAG |
| --- | --- |
| **45** | CCTGATTGAAAGAAATTGCGTAGACCCGAACG  TTTTTTTTAGTAGGTGGTAGAG |
| **46** | ACAGAAATCTTTGAATACCAAGTTCCTTGCTT  TTTTTTTTAGTAGGTGGTAGAG |
| **47** | TTATTAATGCCGTCAATAGATAATCAGAGGTG  TTTTTTTTAGTAGGTGGTAGAG |
| **48** | AGATTAGATTTAAAAGTTTGAGTACACGTAAA  TTTTTTTTAGTAGGTGGTAGAG |
| **49** | AGGCGGTCATTAGTCTTTAATGCGCAATATTA  TTTTTTTTAGTAGGTGGTAGAG |
| **50** | GAATGGCTAGTATTAACACCGCCTCAACTAAT  TTTTTTTTAGTAGGTGGTAGAG |
| **51** | CCGCCAGCCATTGCAACAGGAAAAATATTTTT  TTTTTTTTAGTAGGTGGTAGAG |
| **52** | CCCTCAGAACCGCCACCCTCAGAACTGAGACT  TTTTTTTTAGTAGGTGGTAGAG |
| **53** | CCTCAAGAATACATGGCTTTTGATAGAACCAC  TTTTTTTTAGTAGGTGGTAGAG |
| **54** | TAAGCGTCGAAGGATTAGGATTAGTACCGCCA  TTTTTTTTAGTAGGTGGTAGAG |
| **55** | CACCAGAGTTCGGTCATAGCCCCCGCCAGCAA  TTTTTTTTAGTAGGTGGTAGAG |
| **56** | TCGGCATTCCGCCGCCAGCATTGACGTTCCAG  TTTTTTTTAGTAGGTGGTAGAG |
| **57** | AATCACCAAATAGAAAATTCATATATAACGGA  TTTTTTTTAGTAGGTGGTAGAG |
| **58** | TCACAATCGTAGCACCATTACCATCGTTTTCA  TTTTTTTTAGTAGGTGGTAGAG |
| **59** | ATACCCAAGATAACCCACAAGAATAAACGATT  TTTTTTTTAGTAGGTGGTAGAG |
| **60** | ATCAGAGAAAGAACTGGCATGATTTTATTTTG  TTTTTTTTAGTAGGTGGTAGAG |
| **61** | TTTTGTTTAAGCCTTAAATCAAGAATCGAGAA  TTTTTTTTAGTAGGTGGTAGAG |
| **62** | AGGTTTTGAACGTCAAAAATGAAAGCGCTAAT  TTTTTTTTAGTAGGTGGTAGAG |
| **63** | CAAGCAAGACGCGCCTGTTTATCAAGAATCGC  TTTTTTTTAGTAGGTGGTAGAG |
| **64** | AATGCAGACCGTTTTTATTTTCATCTTGCGGG  TTTTTTTTAGTAGGTGGTAGAG |
| **65** | CATATTTAGAAATACCGACCGTGTTACCTTTT  TTTTTTTTAGTAGGTGGTAGAG |
| **66** | AATGGTTTACAACGCCAACATGTAGTTCAGCT  TTTTTTTTAGTAGGTGGTAGAG |
| **67** | TAACCTCCATATGTGAGTGAATAAACAAAATC  TTTTTTTTAGTAGGTGGTAGAG |
| **68** | AAATCAATGGCTTAGGTTGGGTTACTAAATTT  TTTTTTTTAGTAGGTGGTAGAG |
| **69** | GCGCAGAGATATCAAAATTATTTGACATTATC  TTTTTTTTAGTAGGTGGTAGAG |
| **70** | AACCTACCGCGAATTATTCATTTCCAGTACAT  TTTTTTTTAGTAGGTGGTAGAG |
| **71** | ATTTTGCGTCTTTAGGAGCACTAAGCAACAGT  TTTTTTTTAGTAGGTGGTAGAG |
| **72** | CTAAAATAGAACAAAGAAACCACCAGGGTTAG  TTTTTTTTAGTAGGTGGTAGAG |
| **73** | GCCACGCTATACGTGGCACAGACAACGCTCAT  TTTTTTTTAGTAGGTGGTAGAG |
| **74** | GCGTAAGAGAGAGCCAGCAGCAAAAAGGTTAT  TTTTTTTTAGTAGGTGGTAGAG |
| **75** | GGAAATACCTACATTTTGACGCTCACCTGAAA  TTTTTTTTAGTAGGTGGTAGAG |
| **76** | TATCACCGTACTCAGGAGGTTTAGCGGGGTTT  TTTTTTTTAGTAGGTGGTAGAG |
| **77** | TGCTCAGTCAGTCTCTGAATTTACCAGGAGGT  TTTTTTTTAGTAGGTGGTAGAG |
| **78** | GGAAAGCGACCAGGCGGATAAGTGAATAGGTG  TTTTTTTTAGTAGGTGGTAGAG |
| **79** | TGAGGCAGGCGTCAGACTGTAGCGTAGCAAGG  TTTTTTTTAGTAGGTGGTAGAG |
| **80** | TGCCTTTAGTCAGACGATTGGCCTGCCAGAAT  TTTTTTTTAGTAGGTGGTAGAG |
| **81** | CCGGAAACACACCACGGAATAAGTAAGACTCC  TTTTTTTTAGTAGGTGGTAGAG |
| **82** | ACGCAAAGGTCACCAATGAAACCAATCAAGTT  TTTTTTTTAGTAGGTGGTAGAG |
| **83** | TTATTACGGTCAGAGGGTAATTGAATAGCAGC  TTTTTTTTAGTAGGTGGTAGAG |
| **84** | TGAACAAACAGTATGTTAGCAAACTAAAAGAA  TTTTTTTTAGTAGGTGGTAGAG |
| **85** | CTTTACAGTTAGCGAACCTCCCGACGTAGGAA  TTTTTTTTAGTAGGTGGTAGAG |
| **86** | GAGGCGTTAGAGAATAACATAAAAGAACACCC  TTTTTTTTAGTAGGTGGTAGAG |
| **87** | TCATTACCCGACAATAAACAACATATTTAGGC  TTTTTTTTAGTAGGTGGTAGAG |
| **88** | CCAGACGAGCGCCCAATAGCAAGCAAGAACGC  TTTTTTTTAGTAGGTGGTAGAG |
| **89** | AGAGGCATAATTTCATCTTCTGACTATAACTA  TTTTTTTTAGTAGGTGGTAGAG |
| **90** | TTTTAGTTTTTCGAGCCAGTAATAAATTCTGT  TTTTTTTTAGTAGGTGGTAGAG |
| **91** | TATGTAAACCTTTTTTAATGGAAAAATTACCT  TTTTTTTTAGTAGGTGGTAGAG |
| **92** | TTGAATTATGCTGATGCAAATCCACAAATATA  TTTTTTTTAGTAGGTGGTAGAG |
| **93** | GAGCAAAAACTTCTGAATAATGGAAGAAGGAG  TTTTTTTTAGTAGGTGGTAGAG |
| **94** | TGGATTATGAAGATGATGAAACAAAATTTCAT  TTTTTTTTAGTAGGTGGTAGAG |
| **95** | CGGAATTATTGAAAGGAATTGAGGTGAAAAAT  TTTTTTTTAGTAGGTGGTAGAG |
| **96** | ATCAACAGTCATCATATTCCTGATTGATTGTT  TTTTTTTTAGTAGGTGGTAGAG |
| **97** | CTAAAGCAAGATAGAACCCTTCTGAATCGTCT  TTTTTTTTAGTAGGTGGTAGAG |
| **98** | GCCAACAGTCACCTTGCTGAACCTGTTGGCAA  TTTTTTTTAGTAGGTGGTAGAG |
| **99** | GAAATGGATTATTTACATTGGCAGACATTCTG  TTTTTTTTAGTAGGTGGTAGAG |
| **100** | TTTTTATAAGTATAGCCCGGCCGTCGAG  TTTTTTTTAGTAGGTGGTAGAG |
| **101** | AGGGTTGATTTTATAAATCCTCATTAAATGATATTC  TTTTTTTTAGTAGGTGGTAGAG |
| **102** | ACAAACAATTTTAATCAGTAGCGACAGATCGATAGC  TTTTTTTTAGTAGGTGGTAGAG |
| **103** | AGCACCGTTTTTTAAAGGTGGCAACATAGTAGAAAA  TTTTTTTTAGTAGGTGGTAGAG |
| **104** | TACATACATTTTGACGGGAGAATTAACTACAGGGAA  TTTTTTTTAGTAGGTGGTAGAG |
| **105** | GCGCATTATTTTGCTTATCCGGTATTCTAAATCAGA  TTTTTTTTAGTAGGTGGTAGAG |
| **106** | TATAGAAGTTTTCGACAAAAGGTAAAGTAGAGAATA  TTTTTTTTAGTAGGTGGTAGAG |
| **107** | TAAAGTACTTTTCGCGAGAAAACTTTTTATCGCAAG  TTTTTTTTAGTAGGTGGTAGAG |
| **108** | ACAAAGAATTTTATTAATTACATTTAACACATCAAG  TTTTTTTTAGTAGGTGGTAGAG |
| **109** | AAAACAAATTTTTTCATCAATATAATCCTATCAGAT  TTTTTTTTAGTAGGTGGTAGAG |
| **110** | GATGGCAATTTTAATCAATATCTGGTCACAAATATC  TTTTTTTTAGTAGGTGGTAGAG |
| **111** | AAACCCTCTTTTACCAGTAATAAAAGGGATTCACCAGTCACACGTTTT  TTTTTTTTAGTAGGTGGTAGAG |
| **112** | CCGAAATCCGAAAATCCTGTTTGAAGCCGGAA  TTTTTTTTAGTAGGTGGTAGAG |
| **113** | CCAGCAGGGGCAAAATCCCTTATAAAGCCGGC  TTTTTTTTAGTAGGTGGTAGAG |
| **114** | GCATAAAGTTCCACACAACATACGAAGCGCCA  TTTTTTTTAGTAGGTGGTAGAG |
| **115** | GCTCACAATGTAAAGCCTGGGGTGGGTTTGCC  TTTTTTTTAGTAGGTGGTAGAG |
| **116** | TTCGCCATTGCCGGAAACCAGGCATTAAATCA  TTTTTTTTAGTAGGTGGTAGAG |
| **117** | GCTTCTGGTCAGGCTGCGCAACTGTGTTATCC  TTTTTTTTAGTAGGTGGTAGAG |
| **118** | GTTAAAATTTTAACCAATAGGAACCCGGCACC  TTTTTTTTAGTAGGTGGTAGAG |
| **119** | AGACAGTCATTCAAAAGGGTGAGAAGCTATAT  TTTTTTTTAGTAGGTGGTAGAG |
| **120** | AGGTAAAGAAATCACCATCAATATAATATTTT  TTTTTTTTAGTAGGTGGTAGAG |
| **121** | TTTCATTTGGTCAATAACCTGTTTATATCGCG  TTTTTTTTAGTAGGTGGTAGAG |
| **122** | TCGCAAATGGGGCGCGAGCTGAAATAATGTGT  TTTTTTTTAGTAGGTGGTAGAG |
| **123** | TTTTAATTGCCCGAAAGACTTCAAAACACTAT  TTTTTTTTAGTAGGTGGTAGAG |
| **124** | AAGAGGAACGAGCTTCAAAGCGAAGATACATT  TTTTTTTTAGTAGGTGGTAGAG |
| **125** | GGAATTACTCGTTTACCAGACGACAAAAGATT  TTTTTTTTAGTAGGTGGTAGAG |
| **126** | GAATAAGGACGTAACAAAGCTGCTCTAAAACA  TTTTTTTTAGTAGGTGGTAGAG |
| **127** | CCAAATCACTTGCCCTGACGAGAACGCCAAAA  TTTTTTTTAGTAGGTGGTAGAG |
| **128** | CTCATCTTGAGGCAAAAGAATACAGTGAATTT  TTTTTTTTAGTAGGTGGTAGAG |
| **129** | AAACGAAATGACCCCCAGCGATTATTCATTAC  TTTTTTTTAGTAGGTGGTAGAG |
| **130** | CTTAAACATCAGCTTGCTTTCGAGCGTAACAC  TTTTTTTTAGTAGGTGGTAGAG |
| **131** | TCGGTTTAGCTTGATACCGATAGTCCAACCTA  TTTTTTTTAGTAGGTGGTAGAG |
| **132** | TGAGTTTCGTCACCAGTACAAACTTAATTGTA  TTTTTTTTAGTAGGTGGTAGAG |
| **133** | CCCCGATTTAGAGCTTGACGGGGAAATCAAAA |
| **134** | GAATAGCCGCAAGCGGTCCACGCTCCTAATGA |
| **135** | GAGTTGCACGAGATAGGGTTGAGTAAGGGAGC |
| **136** | GTGAGCTAGTTTCCTGTGTGAAATTTGGGAAG |
| **137** | TCATAGCTACTCACATTAATTGCGCCCTGAGA |
| **138** | GGCGATCGCACTCCAGCCAGCTTTGCCATCAA |
| **139** | GAAGATCGGTGCGGGCCTCTTCGCAATCATGG |
| **140** | AAATAATTTTAAATTGTAAACGTTGATATTCA |
| **141** | GCAAATATCGCGTCTGGCCTTCCTGGCCTCAG |
| **142** | ACCGTTCTAAATGCAATGCCTGAGAGGTGGCA |
| **143** | TATATTTTAGCTGATAAATTAATGTTGTATAA |
| **144** | TCAATTCTTTTAGTTTGACCATTACCAGACCG |
| **145** | CGAGTAGAACTAATAGTAGTAGCAAACCCTCA |
| **146** | GAAGCAAAAAAGCGGATTGCATCAGATAAAAA |
| **147** | TCAGAAGCCTCCAACAGGTCAGGATCTGCGAA |
| **148** | CCAAAATATAATGCAGATACATAAACACCAGA |
| **149** | CATTCAACGCGAGAGGCTTTTGCATATTATAG |
| **150** | ACGAGTAGTGACAAGAACCGGATATACCAAGC |
| **151** | AGTAATCTTAAATTGGGCTTGAGAGAATACCA |
| **152** | GCGAAACATGCCACTACGAAGGCATGCGCCGA |
| **153** | ATACGTAAAAGTACAACGGAGATTTCATCAAG |
| **154** | CAATGACACTCCAAAAGGAGCCTTACAACGCC |
| **155** | AAAAAAGGACAACCATCGCCCACGCGGGTAAA |
| **156** | TGTAGCATTCCACAGACAGCCCTCATCTCCAA |
| **157** | GTAAAGCACTAAATCGGAACCCTAGTTGTTCC |
| **158** | AGTTTGGAGCCCTTCACCGCCTGGTTGCGCTC |
| **159** | AGCTGATTACAAGAGTCCACTATTGAGGTGCC |
| **160** | ACTGCCCGCCGAGCTCGAATTCGTTATTACGC |
| **161** | CCCGGGTACTTTCCAGTCGGGAAACGGGCAAC |
| **162** | CAGCTGGCGGACGACGACAGTATCGTAGCCAG |
| **163** | GTTTGAGGGAAAGGGGGATGTGCTAGAGGATC |
| **164** | CTTTCATCCCCAAAAACAGGAAGACCGGAGAG |
| **165** | AGAAAAGCAACATTAAATGTGAGCATCTGCCA |
| **166** | GGTAGCTAGGATAAAAATTTTTAGTTAACATC |
| **167** | CAACGCAATTTTTGAGAGATCTACTGATAATC |
| **168** | CAATAAATACAGTTGATTCCCAATTTAGAGAG |
| **169** | TCCATATACATACAGGCAAGGCAACTTTATTT |
| **170** | TACCTTTAAGGTCTTTACCCTGACAAAGAAGT |
| **171** | CAAAAATCATTGCTCCTTTTGATAAGTTTCAT |
| **172** | TTTGCCAGATCAGTTGAGATTTAGTGGTTTAA |
| **173** | AAAGATTCAGGGGGTAATAGTAAACCATAAAT |
| **174** | TTTCAACTATAGGCTGGCTGACCTTGTATCAT |
| **175** | CCAGGCGCTTAATCATTGTGAATTACAGGTAG |
| **176** | CGCCTGATGGAAGTTTCCATTAAACATAACCG |
| **177** | TTTCATGAAAATTGTGTCGAAATCTGTACAGA |
| **178** | ATATATTCTTTTTTCACGTTGAAAATAGTTAG |
| **179** | AATAATAAGGTCGCTGAGGCTTGCAAAGACTT |
| **180** | CGTAACGATCTAAAGTTTTGTCGTGAATTGCG |
| **181** | ACCCAAATCAAGTTTTTTGGGGTCAAAGAACG |
| **182** | TGGACTCCCTTTTCACCAGTGAGACCTGTCGT |
| **183** | TGGTTTTTAACGTCAAAGGGCGAAGAACCATC |
| **184** | GCCAGCTGCCTGCAGGTCGACTCTGCAAGGCG |
| **185** | CTTGCATGCATTAATGAATCGGCCCGCCAGGG |
| **186** | ATTAAGTTCGCATCGTAACCGTGCGAGTAACA |
| **187** | TAGATGGGGGGTAACGCCAGGGTTGTGCCAAG |
| **188** | ACCCGTCGTCATATGTACCCCGGTAAAGGCTA |
| **189** | CATGTCAAGATTCTCCGTGGGAACCGTTGGTG |
| **190** | TCAGGTCACTTTTGCGGGAGAAGCAGAATTAG |
| **191** | CTGTAATATTGCCTGAGAGTCTGGAAAACTAG |
| **192** | CAAAATTAAAGTACGGTGTCTGGAAGAGGTCA |
| **193** | TGCAACTAAGCAATAAAGCCTCAGTTATGACC |
| **194** | TTTTTGCGCAGAAAACGAGAATGAATGTTTAG |
| **195** | AAACAGTTGATGGCTTAGAGCTTATTTAAATA |
| **196** | ACTGGATAACGGAACAACATTATTACCTTATG |
| **197** | ACGAACTAGCGTCCAATACTGCGGAATGCTTT |
| **198** | CGATTTTAGAGGACAGATGAACGGCGCGACCT |
| **199** | CTTTGAAAAGAACTGGCTCATTATTTAATAAA |
| **200** | GCTCCATGAGAGGCTTTGAGGACTAGGGAGTT |
| **201** | ACGGCTACTTACTTAGCCGGAACGCTGACCAA |
| **202** | AAAGGCCGAAAGGAACAACTAAAGCTTTCCAG |
| **203** | GAGAATAGCTTTTGCGGGATCGTCGGGTAGCA |
| **204** | ACGTTAGTAAATGAATTTTCTGTAAGCGGAGT |
| **205** | TTTTCGATGGCCCACTACGTAAACCGTC |
| **206** | TATCAGGGTTTTCGGTTTGCGTATTGGGAACGCGCG |
| **207** | GGGAGAGGTTTTTGTAAAACGACGGCCATTCCCAGT |
| **208** | CACGACGTTTTTGTAATGGGATAGGTCAAAACGGCG |
| **209** | GATTGACCTTTTGATGAACGGTAATCGTAGCAAACA |
| **210** | AGAGAATCTTTTGGTTGTACCAAAAACAAGCATAAA |
| **211** | GCTAAATCTTTTCTGTAGCTCAACATGTATTGCTGA |
| **212** | ATATAATGTTTTCATTGAATCCCCCTCAAATCGTCA |
| **213** | TAAATATTTTTTGGAAGAAAAATCTACGACCAGTCA |
| **214** | GGACGTTGTTTTTCATAAGGGAACCGAAAGGCGCAG |
| **215** | ACGGTCAATTTTGACAGCATCGGAACGAACCCTCAG |
| **216** | CAGCGAAAATTTTACTTTCAACAGTTTCTGGGATTTTGCTAAACTTTT |
| **Loop1** | AACATCACTTGCCTGAGTAGAAGAACT |
| **Loop2** | TGTAGCAATACTTCTTTGATTAGTAAT |
| **Loop3** | AGTCTGTCCATCACGCAAATTAACCGT |
| **Loop4** | ATAATCAGTGAGGCCACCGAGTAAAAG |
| **Loop5** | ACGCCAGAATCCTGAGAAGTGTTTTT |
| **Loop6** | TTAAAGGGATTTTAGACAGGAACGGT |
| **Loop7** | AGAGCGGGAGCTAAACAGGAGGCCGA |
| **Loop8** | TATAACGTGCTTTCCTCGTTAGAATC |
| **Loop9** | GTACTATGGTTGCTTTGACGAGCACG |
| **Loop10** | GCGCTTAATGCGCCGCTACAGGGCGC |

1. **References**

[1] G. Bellot, M. A. McClintock, J. J. Chou, W. M. Shih, DNA nanotubes for NMRstructure determination of membrane proteins. *Nat. Protoc*. 2013, 8, 755-770.

[2] E. Stahl, T. G. Martin, F. Praetorius, H. Dietz, Facile and Scalable Preparation of Pure and Dense DNA Origami Solutions. *Angew. Chem. Int. Ed*. 2014, 53, 12735-12740.

[3] a) P. W. Rothemund, Folding DNA to create nanoscale shapes and patterns. Nature 2006, 440, 297-302; b) N. Y. Wong, H. Xing, L. H. Tan, Y. Lu, Nano-Encrypted Morse Code: A Versatile Approach to Programmable and Reversible Nanoscale Assembly and Disassembly. *J. Am. Chem. Soc*. 2013, 135, 2931-2934.
